# Comparing the performance of radiomics, nomograms, machine learning, and large language models in predicting 28-day mortality in severe community-acquired pneumonia patients

**DOI:** 10.3389/fimmu.2025.1679496

**Published:** 2026-01-19

**Authors:** Tingting Lin, Huimin Wan, Yifei Liang, Jie Ming, Jingjing Lu, Zhongliang Guo

**Affiliations:** 1 Fujian Medical University Xiamen Hong’ai Hospital, Xiamen, China; 2 Shanghai East Hospital, School of Medicine, Tongji University, Shanghai, China

**Keywords:** LLMS, machine learning, nomogram, radiomics, SCAP

## Abstract

**Background:**

Severe community-acquired pneumonia (SCAP) is a significant global health challenge due to its high mortality. Despite advances, early diagnosis and effective management remain critical. Tools like radiomics analyze imaging data for risk assessment, while machine learning and nomograms aid in personalized treatment. Large language models (LLMs) enhance clinical decision-making by analyzing data and supporting care strategies. This study integrates these methods to predict 28-day mortality in SCAP patients.

**Methods:**

A cohort of 599 patients diagnosed with severe community-acquired pneumonia (SCAP), including 316 males and 283 females, from Shanghai East Hospital and Xiamen Humanity Hospital were enrolled in this study. High-resolution lung CT scans were used to segment three-dimensional regions of interest, from which 1,050 radiomic features were extracted. The dataset was divided into a training set (80%) and an independent test set (20%), and k-fold cross-validation was applied to optimize model performance. To address class imbalance, the SMOTE oversampling technique was employed. The study integrated radiomics, nomograms, seven machine learning models, and five LLMs to predict the 28-day mortality risk in SCAP patients. SHAP values were utilized to enhance the interpretability of feature contributions. Not only that, this study integrates the prior knowledge provided by LLMs, processed through an embedding layer, with data-driven feature learning in the main network, and dynamically fuses their outputs using a bias network with a gating mechanism, thereby improving the accuracy and interpretability of LLMs in predicting 28-day mortality risk for SCAP patients.

**Results:**

Key predictors of 28-day mortality included inflammatory markers, cytokines, age, CRP, and oxygenation index. Clinical-Radiomics models achieved strong accuracy (AUC 0.92). Machine learning models, particularly XGBoost (AUC 0.90), were highly effective, with SHAP analysis emphasizing radscore’s importance. LLMs like Chatgpt also performed well (AUC 0.78), showcasing the potential of integrating clinical, radiomic, and AI-driven approaches.

**Conclusion:**

This study demonstrates the effectiveness of radiomics, machine learning, and LLMs to predict SCAP outcomes. Models like XGBoost achieved superior accuracy, while SHAP analysis improved interpretability. These advancements highlight the potential for enhanced SCAP prognosis and personalized care strategies.

## Introduction

Community-acquired pneumonia (CAP) poses a significant global health challenge, primarily due to its high incidence and mortality rates. Despite progress in anti-infective treatments and vaccine development, CAP continues to be a leading cause of infection-related deaths worldwide. Severe CAP(SCAP) is one of the main contributors to the rise in global healthcare spending and is a leading cause of sepsis and septic shock, with an extremely high mortality rate. Early diagnosis, effective management, and prompt treatment of SCAP are critical (1–3). To improve SCAP prognosis, various risk assessment models have been developed. Traditional severity scoring systems, such as the Pneumonia Severity Index (PSI) and CURB-65, provide clinical guidelines for determining hospitalization needs (4–9). However, these rule-based methods often fail to fully capture individual patient variations.

In recent years, radiomics is an emerging field in medical imaging that systematically extracts and analyzes high-dimensional features from imaging modalities such as CT, MRI, and PET scans (10–15). These features include characteristics like shape, size, texture, and intensity of lung tissue. By providing a more detailed analysis of imaging data, radiomics is transforming the diagnosis and treatment of severe pneumonia. It detects subtle changes in lung tissue that traditional methods may miss, enabling earlier disease detection and better risk assessment. By quantifying these imaging characteristics, radiomics can accurately classify pneumonia and inform more targeted treatment decisions. Additionally, radiomic analysis can predict the severity and progression of pneumonia, as well as forecast patient outcomes, thereby guiding timely clinical interventions and supporting personalized patient care (16–19).

Nomograms and machine learning each offer unique contributions to medicine. Nomograms are simple, graphical tools that assign weights to clinical variables like age, gender, and medical history to predict disease risk or recurrence, commonly used in cancer prognosis, cardiovascular risk assessment, and chronic disease management. Their main advantage is their ease of use, allowing clinicians to quickly assess risks and make quantitative predictions (20, 21). In contrast, machine learning leverages algorithms to automatically learn from large datasets, excelling in disease diagnosis, medical image analysis, and personalized treatment (22–32). Techniques like convolutional neural networks (CNNs) detect abnormalities in medical images, improving diagnostic accuracy. Machine learning is also pivotal in disease prediction, drug discovery, and clinical decision support systems (CDSS), processing diverse data types to optimize predictive models (33, 34). Nomograms and machine learning are not mutually exclusive alternatives but rather complementary tools in medical prognosis. Nomograms serve as visual representations of regression-based models, allowing clinicians to interpret risk factors easily. If a machine learning algorithm is linear, such as logistic regression, its results can be translated into a nomogram. However, for non-linear ML models (e.g., decision trees, ensemble learning, deep learning), nomograms may not accurately capture their decision-making process.

Beyond radiomics and machine learning, large language models (LLMs) have recently demonstrated remarkable potential in healthcare. LLMs have expanded their role in medicine by extracting valuable information from vast amounts of medical literature, aiding research in drug targets and disease mechanisms while supporting evidence-based clinical decision-making. They analyze unstructured data in electronic medical records (EMRs), helping doctors extract critical information like symptoms and diagnoses, and provide personalized treatment recommendations. In CDSS, LLMs integrate data from various sources to offer tailored diagnostic advice and risk assessments. They are also integral to medical question-answering systems, improving communication between patients and healthcare providers. LLMs enhance multilingual support for global healthcare, assist in personalized treatment based on patient history and genetic data, and contribute to medical education by simulating clinical scenarios and providing case analysis, thus enhancing training efficiency and quality (35–40).

Therefore, this study aims to predict the 28-day mortality rate in SCAP patients by integrating radiomics, nomograms, machine learning, and LLMs.

## Materials and methods

### Subjects

All subjects were selected from Shanghai East Hospital affiliated with Tongji University and Xiamen Humanity Hospital, with the study period extending from July 5, 2022, to April 16, 2025. The study included a total of 599 patients with SCAP(East Hospital affiliated with Tongji University:230, Xiamen Humanity Hospital:369), comprising 316 males and 283 females. Demographic data and clinical information of the participants were collected. The diagnosis of SCAP had to meet established diagnostic criteria (6). The inclusion criteria for the study were: (1) age 18 years and older; (2) diagnosed with community-acquired pneumonia based on clinical symptoms, signs, and radiological examination; (3) requiring hospitalization; (4) having etiological evidence, such as positive pathogen culture or nucleic acid testing; (5) meeting the criteria for severe pneumonia(including a respiratory rate > 30 breaths/min, oxygenation index (PaO2/FiO2) ≤ 250 mmHg, multilobar infiltration, altered mental status and/or disorientation, blood urea nitrogen ≥ 7.14 mmol/L, systolic blood pressure< 90 mmHg, leukopenia (WBC)< 4*10^9/L, thrombocytopenia (PLT)< 100*10^9/L, and hypothermia< 36°C). Exclusion criteria included: (1) under 18 years of age; (2) having received more than 48 hours of antibiotic treatment; (3) presence of immunodeficiency or immunosuppressive state, such as HIV infection, post-organ transplantation, etc. (4) the image has significant artifacts, affecting analysis. This study was approved by the Ethics Committee of Shanghai East Hospital and Xiamen Humanity Hospital (Ethics Committee Number:<2022>Research Review No. (216) and HAXN-MEC-20250416-029-01).

### Workflow of the study

**Figure 1:**
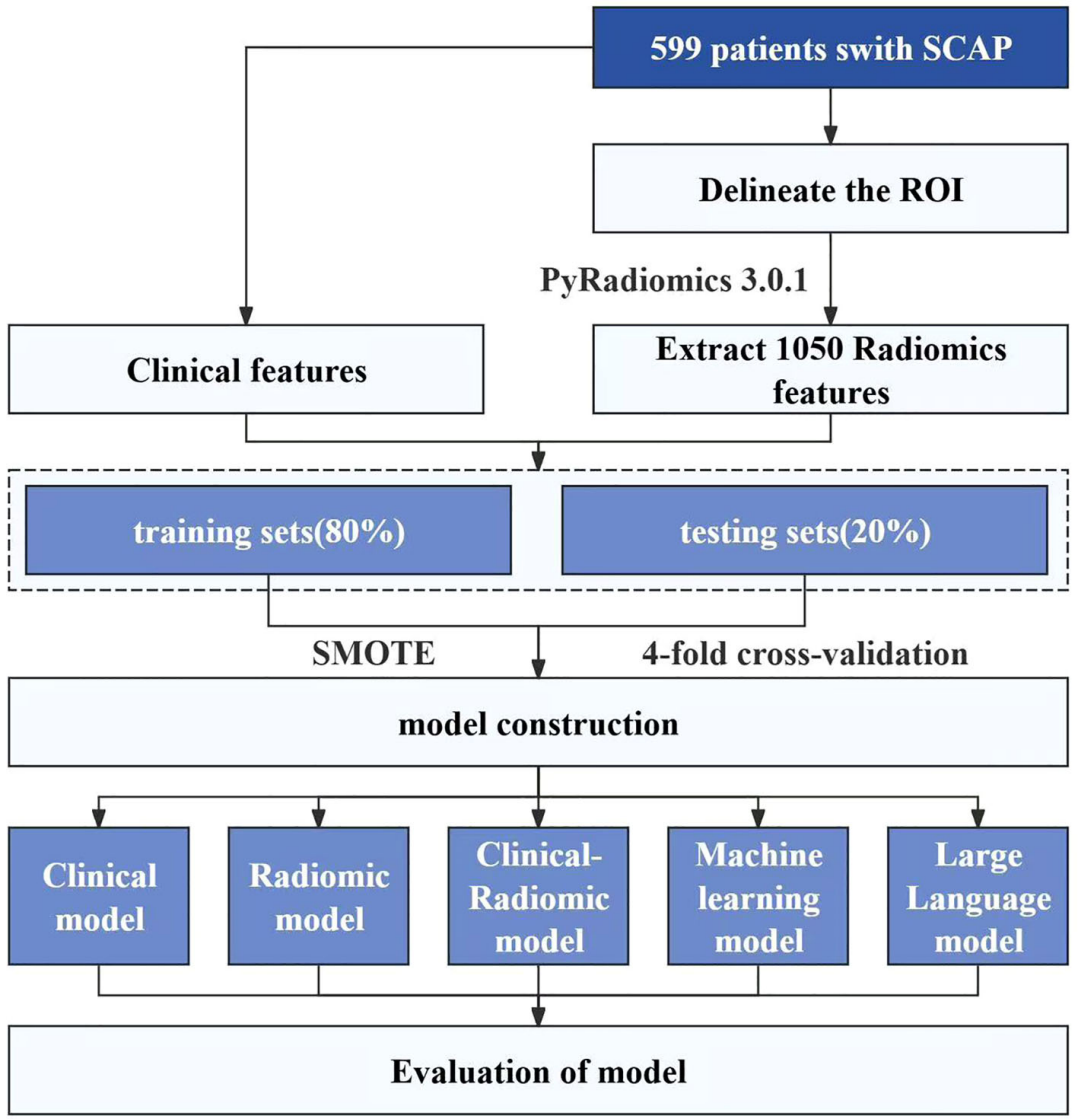
Workflow of model construction and evaluation for SCAP patients.

This flowchart (**Figure 1**) illustrates the experimental process of a study based on clinical and radiomics data from 599 SCAP patients. A total of 1,050 radiomics features were extracted and integrated with clinical features for modeling. The data were balanced using the SMOTE method and underwent four-fold cross-validation. Five models were constructed and evaluated: a clinical model, a radiomics model, a clinical-radiomics combined model, a machine learning model, and a large language model.

### Instruments and methods

Images were acquired using a Philips 128-slice spiral CT. The patient was positioned supine with head first, and a continuous CT scan was performed at the end of inspiration with breath-holding. The scan covered the entire lung field, with a tube voltage of 120 kV and automatic tube current adjustment using care Dose. The slice thickness and interslice gap were 1 mm, and the image matrix size was 512 × 512.

### Delineating the region of interest

**Figure 2:**
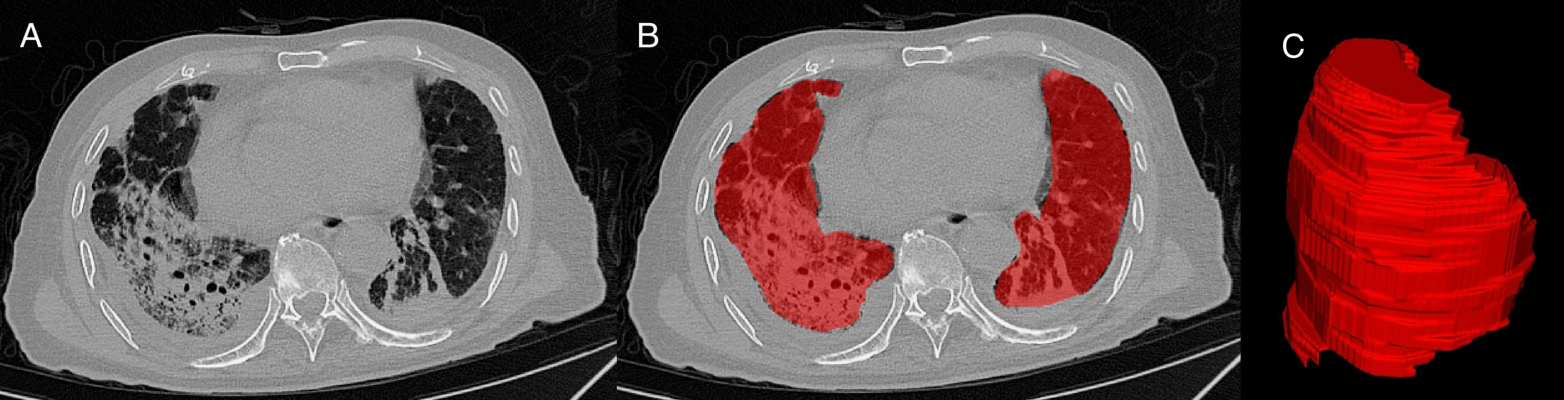
CT-based lung lesion segmentation and 3D reconstruction. **(A)** Original CT image. **(B)** Manual segmentation of the region of interest (ROI) on CT images (highlighted in red). **(C)** Three-dimensional reconstruction of the ROI.

CT DICOM files of 599 patients were imported into the open-source image segmentation software ITK-SNAP. Lesions were manually delineated on each slice using the software’s tools. Subsequently, a 3D ROI (Region of Interest) was extracted from these areas (**Figure 2**).

### Evaluate consistency

First, radiologist A manually delineated the ROI layer by layer for all case samples. Meanwhile, imaging data from 30 randomly selected patients were segmented by another radiologist, B. To further assess intra-observer consistency, radiologist A repeated the ROI delineation for all cases, with at least a one-month interval between the two delineations. Both radiologists were unaware of the pathological results of the lesions during the delineation process. This study used interclass or intraclass correlation coefficients (ICCs) to evaluate the consistency of feature extraction between and within observers, with ICCs > 0.75 indicating good consistency. Meanwhile, radiomic features with ICCs less than or equal to 0.75 were excluded from the analysis.

### Extract radiomics features

We utilized the open-source software Python 3.6.3 (https://www.python.org/) with the PyRadiomics 3.0.1 package to extract radiomic features from regions of interest. The types of radiomic features extracted included: First order, GLCM, GLDM, GLSZM, NGTDM, Shape3d, Shape2d, Log Kernel Size (3,4), and Wavelet-Based Features. In total, 1050 features were extracted.

### Model training and validation

**Figure 3:**
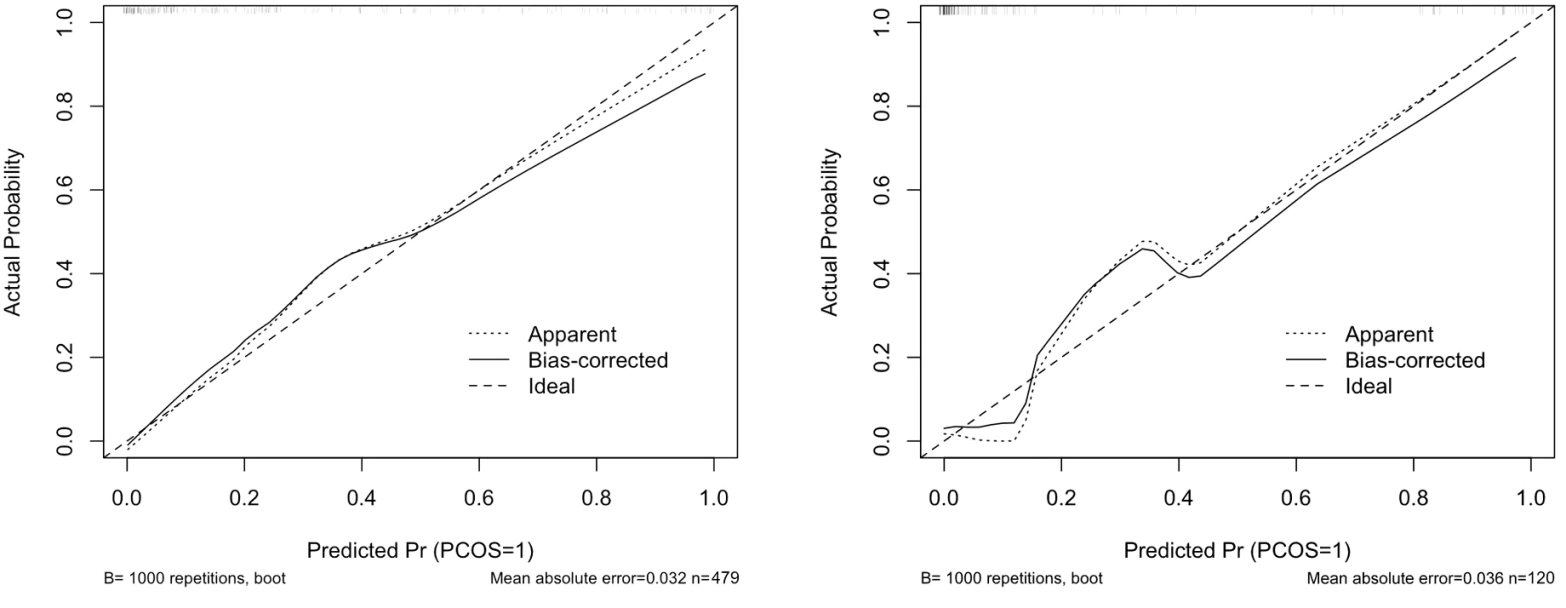
Calibration curves for training and testing sets.

In this study, we implemented a rigorous model training and validation process to ensure the robustness and reliability of our classification models. The dataset was partitioned into training (80%) and testing (20%) sets. (**Figure 3**) This division aligns with best practices in machine learning, allowing for comprehensive training, thorough model tuning, and reliable evaluation of model performance. The training set was utilized to fit various classification models, including logistic regression and random forests. To optimize model parameters and mitigate overfitting, we employed 4-fold cross-validation. Performance metrics such as accuracy, precision, recall, and F1 score were calculated and averaged over the 4 iterations to provide a robust estimate of model performance. To address potential issues of class imbalance, techniques like SMOTE (Synthetic Minority Over-sampling Technique) were applied (41). The final model based on its performance selection in cross validation and validation set adjustment. The validation set, separate from the training data, was used for fine-tuning model hyperparameters. This systematic approach ensures the selection of an effective classifier for our predictive task, underlining the model’s practical utility.

### Assessment of models

This paper employs widely used evaluation metrics from statistics and machine learning to offer various perspectives on model performance. These metrics include Accuracy, F1 score, AUC score, Sensitivity, Specificity, Positive Predictive Value, and Negative Predictive Value. These metrics are especially important in imbalanced datasets, where relying solely on accuracy might not provide a comprehensive assessment of the model’s performance.

### Model explainability

SHAP (Shapley Additive exPlanations) values are a method based on Shapley values from game theory, used to explain predictions of machine learning models (42). They offer an intuitive explanation of the model’s decision-making by quantifying the contribution level of each feature to the model’s prediction. Each SHAP value signifies the contribution of a specific feature value relative to a baseline or average prediction. This approach is applicable to various types of machine learning models and provides a consistent way of interpretation (43).

### Application of large language models in predicting 28-day mortality for SCAP patients

**Figure 4:**
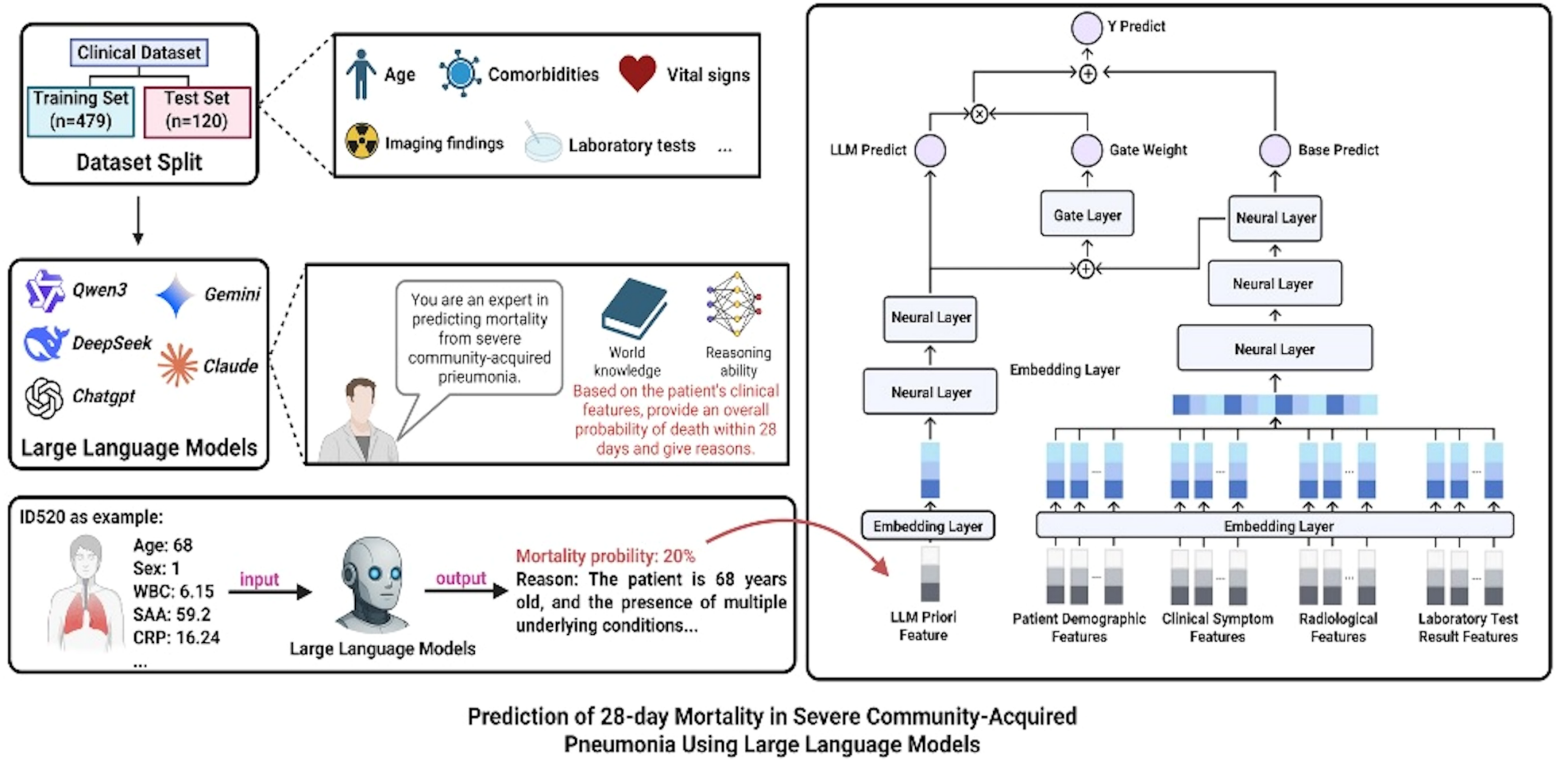
Framework of the LLM-integrated predictive system for 28-day mortality risk in SCAP patients.

We developed a Knowledge-Data Dual-Driven Framework to construct a predictive system for the 28-day mortality risk in patients with SCAP. In this framework, we incorporate the Large Language Model not merely as a feature extractor, but as a reasoned prior provider. Technically, we implement a Gating Mechanism within a Bias Network that dynamically regulates the contribution of the LLM’s prior probability against the data-driven features extracted from radiomics and clinical variables. Based on five mainstream large language models (LLMs)—Qwen3, DeepSeek, Gemini, Claude, and ChatGPT. The research begins with a systematic division of the clinical dataset, randomly splitting the data of 599 SCAP patients into a training set (479 cases) and an independent test set (120 cases) at a 4:1 ratio to ensure the reliability of model evaluation. To guarantee the comparability and consistency of results across different models, a unified role context was established: “ You are an expert in the field of severe community-acquired pneumonia, and you are currently participating in an assessment aimed at predicting the risk of death within 28 days after onset in patients with severe community-acquired pneumonia. In this process, you need to thoroughly understand and deeply analyze a range of clinical features and risk factors associated with patient outcomes.” Under this framework, all models were given the same task instruction: “Your task is to estimate the patient’s probability of mortality based on their clinical features and provide the reasoning behind your assessment. The prediction results will be used directly for the patient’s diagnosis, and any errors in prediction may result in significant harm or even death.” To ensure the reproducibility and stability of these outputs, we explicitly set the temperature parameter to 0 for all LLMs. This configuration minimizes the stochasticity inherent in the generation process, ensuring deterministic responses essential for reliable clinical risk assessment. Additionally, the maximum output token limit was set to 500 to accommodate concise reasoning and probability estimation. Preliminary validation indicated that this fixed ‘Role-Task-Context’ structure, combined with a zero-temperature setting, provided the most consistent medical reasoning compared to variable prompts or higher entropy settings. This design not only reinforces the models’ role immersion in the specific medical domain but also effectively guides them to leverage their existing world knowledge and reasoning capabilities, thereby enabling more accurate interpretation and analysis of patients’ multidimensional clinical characteristics. During execution, each LLM conducts an in-depth analysis of key variables such as the patient’s age, underlying diseases, vital signs, laboratory tests, and imaging findings. By integrating their learned knowledge from vast medical literature, clinical guidelines, and expert experience, the models quantitatively assess individual mortality risk and output specific mortality probabilities along with explanatory reasoning. The input layer includes predictions of patient mortality probabilities from LLMs (LLM Priori Feature), patient demographic features, clinical symptom features, radiological features, and laboratory test results. These input features are converted into vector representations via an embedding layer for processing by the neural network. In the main neural network (Main Network), multiple neural layers learn complex interactions among input features, extracting features and performing nonlinear transformations through multilayer perceptrons to generate a base prediction result (Base Predict). Technically, the fusion process operates as follows: First, the numerical mortality probability is extracted from the LLM’s output to serve as the Prior Knowledge ( $P_{LLM}$). Simultaneously, the Main Network processes the vectorized clinical and radiomics features to generate a data-driven base prediction ( $P_{Data}$). To integrate these, the Bias Network computes a dynamic scalar ‘Gate Weight’ ( G) via a sigmoid activation function based on the input feature distribution. The final mortality risk ( $Y_{final}$) is computed as a dynamically weighted sum: $Y_{final}$ = G · $P_{Data}$ + (1 - G) · $p_{LLM}$. This mechanism allows the model to adaptively shift its focus between data-driven patterns and knowledge-based priors depending on the specific complexity of each patient case. Meanwhile, in the biased network, the gate layer calculates gate weights based on specific circumstances, dynamically adjusting the fusion ratio between the output of the main network and the LLM predictions. This allows the final prediction result (Y Predict) to effectively combine data-driven pattern recognition with expert knowledge-based reasoning. This design ensures that the system can leverage data-driven approaches while incorporating expert knowledge, thereby improving the accuracy and reliability of predictions (**Figure 4**).

### Statistics and software

All statistical analysis processes in this study were performed using Python (version 3.6.3), R (version 3.6.1), and ITK-SNAP (version 3.8.0).

## Result

### Demographic characteristics and clinical information

53 features were included in this study, and a comprehensive analysis of clinical and biochemical parameters between the survival and death groups of SCAP patients was conducted, revealing significant differences and providing insights into potential predictive factors for mortality. This study included a total of 599 patients (East Hospital affiliated with Tongji University:230, Xiamen Humanity Hospital:369), of whom 316 were male and 283 were female. There were 417 survivors and 182 deaths. The univariate analysis of data from 599 patients revealed significant differences between the death and survival groups across several key indicators. Specifically, the median age in the death group was 76 years, significantly higher than the 72 years observed in the survival group (P<0.001). Although there was no marked difference in the percentage of neutrophils between the two groups, white blood cell counts and absolute neutrophil values were higher in the deceased group with statistical significance. Furthermore, inflammatory markers such as C-reactive protein (CRP) and procalcitonin (PCT) were markedly elevated in the death group (P<0.001), suggesting that higher levels of inflammation may be associated with adverse outcomes. Serum amyloid A (SAA), IL-6, IL-17 cytokines, and D-dimer levels were also significantly higher in the death group (P<0.001), indicating that activation of the coagulation system might be a factor in disease progression. Significant differences in respiratory rate and oxygenation index between the two groups (P<0.001) indicated that deterioration in respiratory function is linked to higher mortality rates. In terms of chronic diseases, the proportions of coronary heart disease and hypertension were significantly higher in the death group compared to the survival group (P<0.001), highlighting the impact of underlying health conditions on outcomes. Lastly, the incidence of fever and cough was higher in the death group (P = 0.013 and P = 0.002 respectively), further suggesting that these symptoms may be indicative of more severe illness (**Table 1**).

**Table 1:** Univariate analysis.

Variables	Total (n = 599)	Survival (n = 416)	Death (n = 183)	Statistic	*P*
AGE, M (Q_1_, Q_3_)	74.000 (68.000, 82.000)	72.000 (65.750, 80.000)	76.000 (70.000, 82.500)	Z=-3.974	<.001
WBC, M (Q_1_, Q_3_)	7.780 (5.335, 11.140)	7.465 (4.995, 10.607)	8.170 (5.755, 12.090)	Z=-2.457	0.014
Percentage Neutrophils, M (Q_1_, Q_3_)	57.360 (50.005, 63.890)	57.300 (50.332, 64.307)	57.440 (49.175, 63.370)	Z=-0.780	0.435
Neutrophil, M (Q_1_, Q_3_)	4.500 (2.880, 6.305)	4.375 (2.795, 6.085)	4.760 (3.150, 6.610)	Z=-2.007	0.045
Percentage Lymphocytes, M (Q_1_, Q_3_)	29.820 (23.355, 36.875)	29.760 (23.030, 36.848)	30.190 (23.800, 36.950)	Z=-0.016	0.987
Lymphocytes, M (Q_1_, Q_3_)	2.220 (1.415, 3.345)	2.110 (1.377, 3.237)	2.330 (1.590, 3.635)	Z=-2.267	0.023
SAA, M (Q_1_, Q_3_)	135.080 (20.450, 257.010)	96.540 (9.285, 218.212)	206.270 (93.885, 264.065)	Z=-6.500	<.001
CRP, M (Q_1_, Q_3_)	37.790 (8.040, 87.170)	34.490 (4.025, 76.670)	47.530 (24.970, 121.165)	Z=-4.323	<.001
PCT, M (Q_1_, Q_3_)	0.772 (0.248, 3.205)	0.772 (0.324, 4.122)	0.734 (0.111, 1.310)	Z=-4.048	<.001
IL-1β, M (Q_1_, Q_3_)	2.500 (1.270, 5.360)	2.500 (1.330, 5.360)	2.500 (1.270, 5.125)	Z=-3.937	<.001
IL-2, M (Q_1_, Q_3_)	2.500 (1.340, 5.190)	2.500 (1.340, 5.190)	2.500 (1.280, 5.245)	Z=-1.564	0.118
IL-4, M (Q_1_, Q_3_)	2.500 (1.340, 4.960)	2.500 (1.340, 4.960)	2.500 (1.330, 4.775)	Z=-1.710	0.087
IL-5, M (Q_1_, Q_3_)	2.490 (1.370, 2.520)	2.500 (1.370, 2.520)	2.300 (1.335, 2.500)	Z=-2.227	0.026
IL-6, M (Q_1_, Q_3_)	27.790 (2.990, 99.145)	19.250 (2.820, 97.152)	38.260 (9.805, 100.440)	Z=-3.258	0.001
IL-8, M (Q_1_, Q_3_)	34.290 (9.410, 68.595)	33.460 (6.475, 67.647)	36.060 (17.130, 71.370)	Z=-2.204	0.028
IL-10, M (Q_1_, Q_3_)	3.620 (1.320, 7.510)	3.635 (1.380, 6.962)	3.560 (1.320, 8.030)	Z=-0.116	0.907
IL-12P70, M (Q_1_, Q_3_)	2.500 (1.270, 4.790)	2.500 (1.270, 4.790)	2.500 (1.270, 4.715)	Z=-2.759	0.006
IL-17, M (Q_1_, Q_3_)	5.770 (1.660, 14.195)	7.180 (2.500, 15.930)	3.160 (1.640, 10.870)	Z=-4.533	<.001
IFNα, M (Q_1_, Q_3_)	2.600 (1.350, 6.570)	2.870 (1.770, 7.550)	2.500 (1.350, 5.625)	Z=-3.199	0.001
IFNγ, M (Q_1_, Q_3_)	2.720 (1.810, 4.965)	2.980 (2.120, 5.262)	2.500 (1.360, 4.435)	Z=-2.701	0.007
TNFα, M (Q_1_, Q_3_)	2.500 (1.350, 7.330)	2.895 (1.350, 7.330)	2.500 (1.260, 5.490)	Z=-2.673	0.008
PaO2, M (Q_1_, Q_3_)	93.120 (80.695, 105.300)	94.065 (81.683, 106.040)	90.220 (79.220, 103.835)	Z=-1.389	0.165
SaO2, M (Q_1_, Q_3_)	97.880 (95.180, 100.200)	97.900 (95.300, 99.948)	97.730 (94.250, 100.470)	Z=-0.029	0.977
PaCO2, M (Q_1_, Q_3_)	38.790 (34.635, 44.095)	39.500 (35.195, 44.895)	38.500 (33.530, 40.465)	Z=-3.310	<.001
BE, M (Q_1_, Q_3_)	0.700 (-1.500, 2.900)	0.100 (-1.925, 2.300)	2.100 (-0.450, 3.700)	Z=-4.558	<.001
cTnT, M (Q_1_, Q_3_)	0.017 (0.008, 0.029)	0.014 (0.007, 0.027)	0.019 (0.012, 0.033)	Z=-3.831	<.001
D-dimer, M (Q_1_, Q_3_)	1.210 (0.310, 5.115)	0.830 (0.310, 2.980)	2.940 (0.875, 13.135)	Z=-7.224	<.001
AST, M (Q_1_, Q_3_)	27.000 (23.000, 37.000)	27.500 (21.000, 38.000)	27.000 (24.000, 36.000)	Z=-1.725	0.085
ALT, M (Q_1_, Q_3_)	24.000 (19.000, 35.000)	24.000 (18.000, 35.000)	24.000 (22.000, 33.000)	Z=-1.097	0.273
Lac, M (Q_1_, Q_3_)	1.900 (1.500, 2.600)	1.900 (1.575, 2.700)	2.000 (1.500, 2.500)	Z=-0.098	0.922
Cr, M (Q_1_, Q_3_)	69.000 (57.000, 88.000)	73.000 (59.000, 88.000)	63.000 (51.500, 92.000)	Z=-1.904	0.057
Oxygenation Index, M (Q_1_, Q_3_)	266.000 (266.000, 266.100)	266.000 (266.000, 266.100)	266.000 (266.000, 266.000)	Z=-4.774	<.001
Respiratory rate, M (Q_1_, Q_3_)	18.000 (18.000, 18.000)	18.000 (18.000, 18.000)	18.000 (18.000, 19.000)	Z=-5.836	<.001
Systolic blood pressure, M (Q_1_, Q_3_)	116.000 (115.500, 116.000)	116.000 (115.500, 116.000)	116.000 (115.000, 116.000)	Z=-0.997	0.319
Hospitalization Days, M (Q_1_, Q_3_)	14.000 (12.000, 15.000)	14.000 (12.750, 15.000)	14.000 (12.000, 14.000)	Z=-0.310	0.757
Gamma Globulin Days, M (Q_1_, Q_3_)	0.000 (0.000, 0.000)	0.000 (0.000, 0.000)	0.000 (0.000, 3.000)	Z=-7.092	<.001
Radscore, M (Q_1_, Q_3_)	-1.114 (-1.767, -0.189)	-1.328 (-2.310, -0.994)	0.403 (-0.301, 1.058)	Z=-16.709	<.001
Sex, n(%)				χ²=0.969	0.325
0	283 (47.245)	191 (45.913)	92 (50.273)		
1	316 (52.755)	225 (54.087)	91 (49.727)		
Hormone, n(%)				χ²=3.295	0.069
0	171 (28.548)	128 (30.769)	43 (23.497)		
1	428 (71.452)	288 (69.231)	140 (76.503)		
Coronary heart disease, n(%)				χ²=30.788	<.001
0	473 (78.965)	303 (72.837)	170 (92.896)		
1	126 (21.035)	113 (27.163)	13 (7.104)		
Chronic bronchitis, n(%)				χ²=2.403	0.121
0	529 (88.314)	373 (89.663)	156 (85.246)		
1	70 (11.686)	43 (10.337)	27 (14.754)		
COPD, n(%)				χ²=0.556	0.456
0	566 (94.491)	395 (94.952)	171 (93.443)		
1	33 (5.509)	21 (5.048)	12 (6.557)		
Atrial fibrillation, n(%)				χ²=0.225	0.636
0	569 (94.992)	394 (94.712)	175 (95.628)		
1	30 (5.008)	22 (5.288)	8 (4.372)		
Diabetes, n(%)				χ²=1.390	0.238
0	439 (73.289)	299 (71.875)	140 (76.503)		
1	160 (26.711)	117 (28.125)	43 (23.497)		
Hypertension, n(%)				χ²=45.758	<.001
0	247 (41.235)	134 (32.212)	113 (61.749)		
1	352 (58.765)	282 (67.788)	70 (38.251)		
Fever, n(%)				χ²=6.137	0.013
0	50 (8.347)	27 (6.490)	23 (12.568)		
1	549 (91.653)	389 (93.510)	160 (87.432)		
Cough, n(%)				χ²=9.403	0.002
0	14 (2.337)	4 (0.962)	10 (5.464)		
1	585 (97.663)	412 (99.038)	173 (94.536)		
Dyspnea, n(%)				χ²=0.160	0.689
0	538 (89.816)	375 (90.144)	163 (89.071)		
1	61 (10.184)	41 (9.856)	20 (10.929)		
Disturbance of consciousness, n(%)				χ²=2.205	0.138
0	593 (98.998)	414 (99.519)	179 (97.814)		
1	6 (1.002)	2 (0.481)	4 (2.186)		
Multiple lobar infiltration, n(%)				χ²=1.341	0.247
0	453 (75.626)	309 (74.279)	144 (78.689)		
1	146 (24.374)	107 (25.721)	39 (21.311)		
Atelectasis of the lungs, n(%)				χ²=0.528	0.468
0	574 (95.826)	397 (95.433)	177 (96.721)		
1	25 (4.174)	19 (4.567)	6 (3.279)		
Pleural effusion, n(%)				χ²=0.020	0.887
0	561 (93.656)	390 (93.750)	171 (93.443)		
1	38 (6.344)	26 (6.250)	12 (6.557)		

**Table 2** summarizes the regression coefficients (β), standard errors (S.E.), Z-values, P-values, odds ratios (OR), and their 95% confidence intervals (CI) from the logistic regression analysis. An increase in age by one year is associated with an increased risk of adverse outcomes (OR: 1.053, 95% CI: 1.014–1.094, P = 0.007). Respiratory rate and oxygenation index also showed significant effects, corresponding to higher risk (OR: 1.815, 95% CI: 1.390–2.370, P<0.001) and a protective effect (OR: 1.015, 95% CI: 1.007–1.023, P<0.001), respectively. Notably, the presence of coronary heart disease (OR: 0.281, 95% CI: 0.113–0.700, P = 0.006) and hypertension (OR: 0.316, 95% CI: 0.158–0.633, P = 0.001) were linked to lower risks, which may be due to control group selection or differences in treatment effects. In addition, radscore (OR: 7.751, 95% CI: 4.933–12.177, P<0.001) demonstrate significant associations, highlighting their importance in predicting patient outcomes.

**Table 2:** Logistic regression.

Variables	β	S.E	Wald	*P*	OR (95%CI)
AGE	0.052	0.019	2.701	0.007	1.053 (1.014 ~ 1.094)
WBC	-0.087	0.348	-0.251	0.802	0.916 (0.463 ~ 1.813)
Neutrophil	0.068	0.392	0.174	0.862	1.071 (0.497 ~ 2.308)
Lymphocytes	0.053	0.443	0.119	0.905	1.054 (0.443 ~ 2.510)
SAA	0.002	0.002	0.998	0.318	1.002 (0.998 ~ 1.005)
CRP	-0.001	0.003	-0.352	0.725	0.999 (0.992 ~ 1.005)
IL-1β	-0.114	0.094	-1.206	0.228	0.892 (0.742 ~ 1.074)
PCT	-0.058	0.061	-0.954	0.340	0.944 (0.838 ~ 1.063)
IL-17	-0.013	0.019	-0.714	0.475	0.987 (0.951 ~ 1.024)
IFN α	-0.086	0.069	-1.238	0.216	0.918 (0.801 ~ 1.051)
IFN γ	0.048	0.087	0.554	0.580	1.049 (0.885 ~ 1.244)
TNF α	-0.150	0.069	-2.170	0.030	0.861 (0.752 ~ 0.986)
SaO2	-0.020	0.047	-0.437	0.662	0.980 (0.894 ~ 1.074)
PaCO2	-0.049	0.027	-1.776	0.076	0.952 (0.902 ~ 1.005)
BE	0.059	0.064	0.920	0.357	1.061 (0.935 ~ 1.203)
D-dimer	0.038	0.027	1.421	0.155	1.039 (0.986 ~ 1.095)
Respiratory rate	0.596	0.136	4.378	<.001	1.815 (1.390 ~ 2.370)
Oxygenation Index	0.015	0.004	3.664	<.001	1.015 (1.007 ~ 1.023)
Systolic blood pressure	-0.071	0.028	-2.505	0.012	0.932 (0.881 ~ 0.985)
Gamma Globulin Days	0.266	0.124	2.155	0.031	1.305 (1.024 ~ 1.663)
Radscore	2.048	0.230	8.884	<.001	7.751 (4.933 ~ 12.177)
Coronary heart disease					
0					1.000 (Reference)
1	-1.269	0.465	-2.727	0.006	0.281 (0.113 ~ 0.700)
Hypertension					
0					1.000 (Reference)
1	-1.152	0.354	-3.253	0.001	0.316 (0.158 ~ 0.633)
Cough					
0					1.000 (Reference)
1	-1.990	1.674	-1.189	0.235	0.137 (0.005 ~ 3.636)
Fever					
0					1.000 (Reference)
1	-1.269	0.701	-1.810	0.070	0.281 (0.071 ~ 1.111)

### Radiomic feature selection

**Figure 5:**
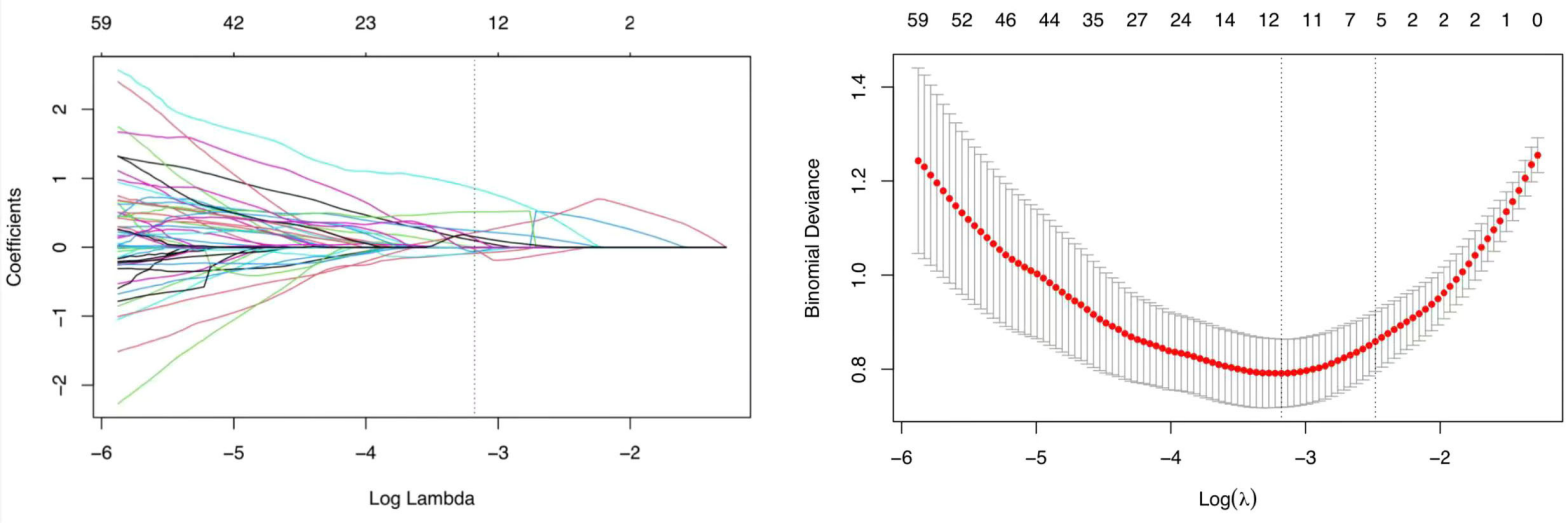
Lasso regression path diagram and Lasso regression cross validation error path diagram.

**Figure 6:**
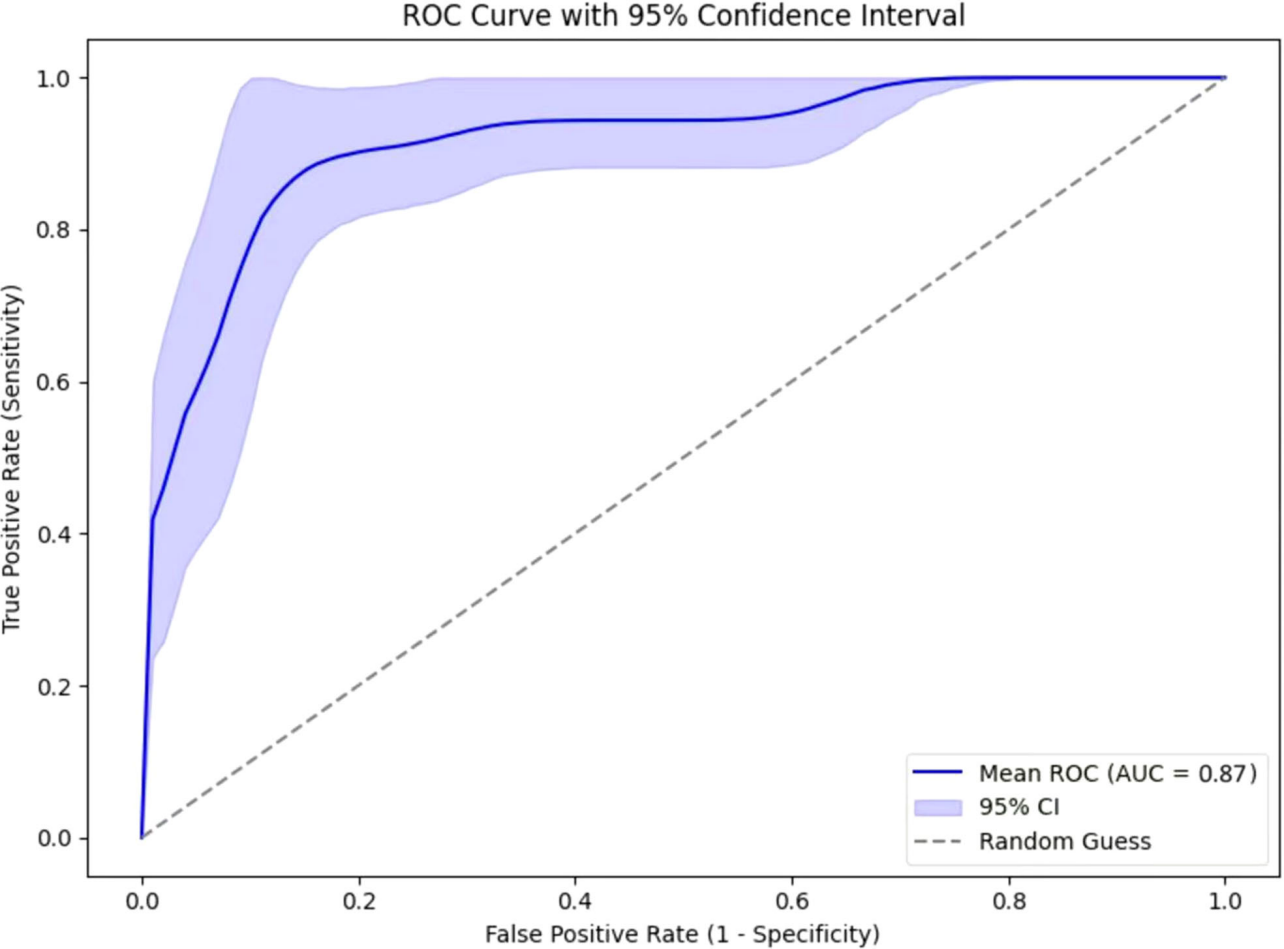
ROC curves of radiomics-only model.

A total of 1050 radiomic features were extracted from 599 SCAP CT scans. By using the U-test and Lasso regression, 12 features were selected (**Figure 5**, **Table 3**), and the radscore was calculated as follows:-1.087672 + 9.669283xoriginal_shape_Flatness+2.135276x original_g1r1m_RunLengthNonUniformity + (-9.014526) x wavelet.LLH_firstorder_Median + (-6.970532) x wavelet.LHL_firstorder_Median + 1.249196 x wavelet.LHL_glcm_clustershade + (-2.792732)xwavelet.LHL_glcm_JointAverage+(-6.051102)x wavelet.LHL_g1dm_HighGrayLevelEmphasis+1.003973xwavelet.LHL_g1dm_LowGrayLevelEmphasis+2.404839xwavelet.HLL_g1szm_SizeZoneNonUniformity+5.155273 x wavelet.LLL_firstorder_Mean + 8.531983 x wavelet.LLL_g1r1m_RunLengthNonuniformity + 1.516815 x wavelet.LLL_ngtdm_contrast. The ROC curve for the radiomics-only model is presented in **Figure 6**. The model achieved an AUC of 0.87, indicating a strong predictive capability of radiomics features in this context. The ROC curve suggests that radiomics-derived features provide meaningful discrimination ability.

**Table 3:** Radiomic features.

Radiomic features	Coefficient
original_shape_Flatness	9.669283E-02
original_g1r1m_RunLengthNonUniformity	2.135276E-02
wavelet.LLH_firstorder_Median	-9.014526E-02
wavelet.LHL_firstorder_Median	-6.970532E-02
wavelet.LHL_g1cm_Clustershade	1.249196E-01
wavelet.LHL_g1cm_JointAverage	-2.792732E-02
wavelet.LHL_g1dm_HighGrayLevelEmphasis	-6.051102E-02
wavelet.LHL_gldm_LowGrayLevelEmphasis	1.003973E-06
wavelet.HLL_g1szm_SizeZoneNonUniformity	2.404839E-01
wavelet.LLL_firstorder_Mean	5.155273E-01
wavelet.LLL_g1r1m_RunLengthNonUniformity	8.531983E-01
wavelet.LLL_ngtdm_contrast	1.516815E-01
(Intercept)	-1.087672E+00

### Key predictive indicators and insights from spearman correlation analysis in disease assessment

**Figure 7:**
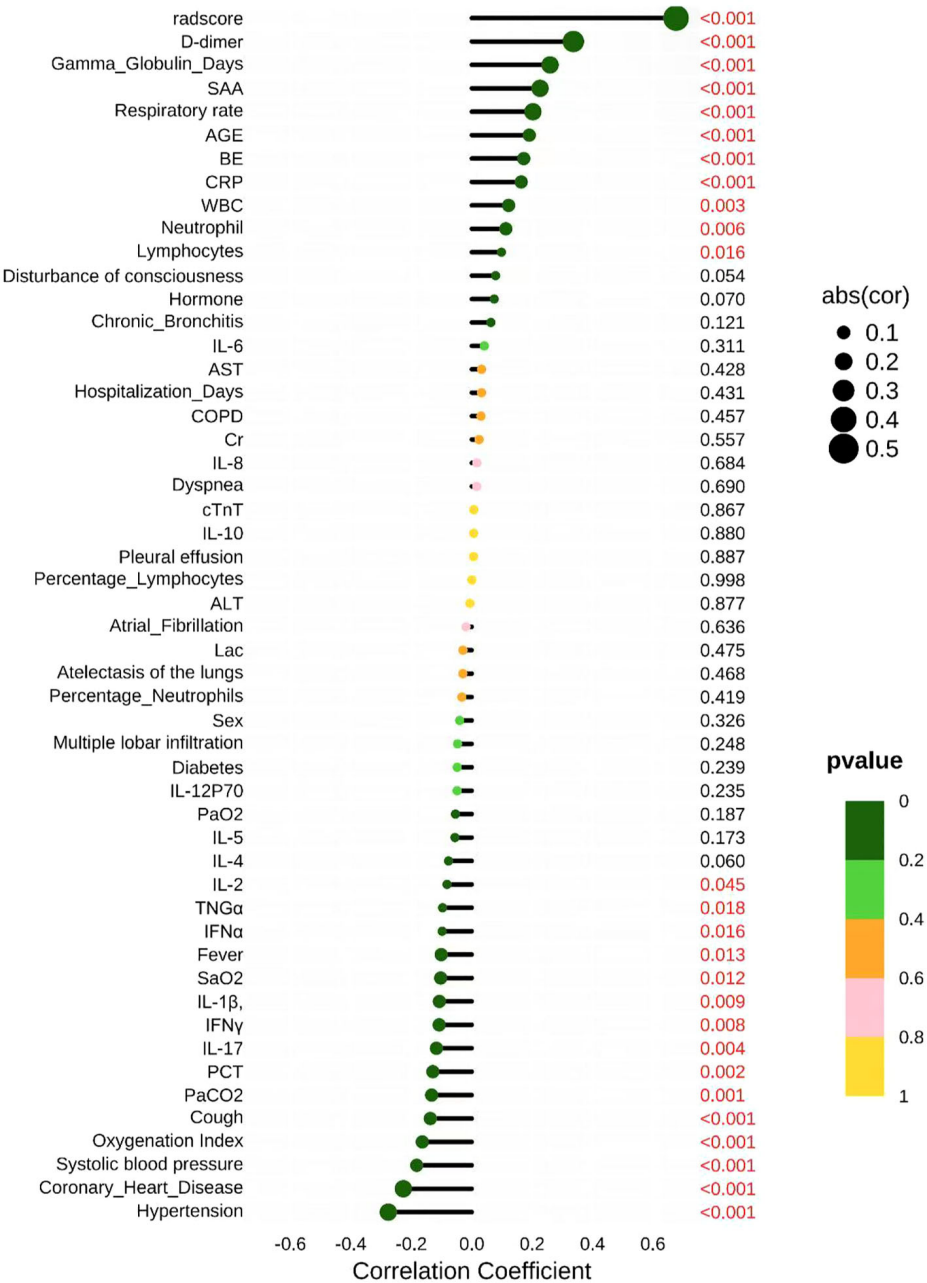
Pearson coefficient plot.

In the Spearman correlation analysis, several variables showed statistical significance. Among them, the highly significant variables (P< 0.001) included: radscore, D-dimer, SAA Respiratory rate, AGE, BE, and CRP. Notably, variables such as Chronic_Bronchitis, IL-6, AST, COPD, and Cr exhibited weaker correlations or non-significant P-values. Furthermore, the Oxygenation Index, Coronary Heart Disease, and Hypertension also demonstrated highly significant associations (P< 0.001), indicating strong statistical relationships between these variables and the study outcomes (**Figure 7**).

### Construction and evaluation of the nomogram model

**Figure 8:**
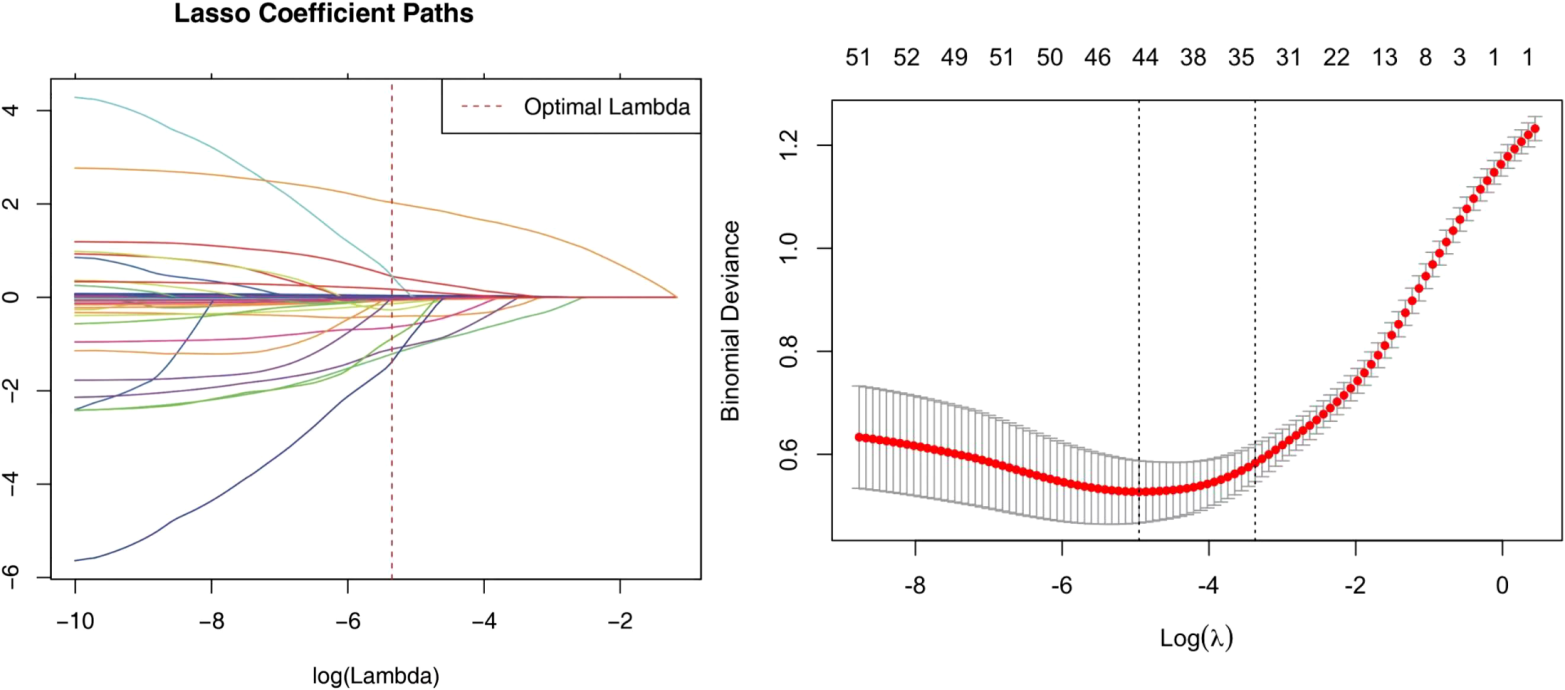
Lasso regression path diagram and Lasso regression cross validation error path diagram.

**Figure 9:**
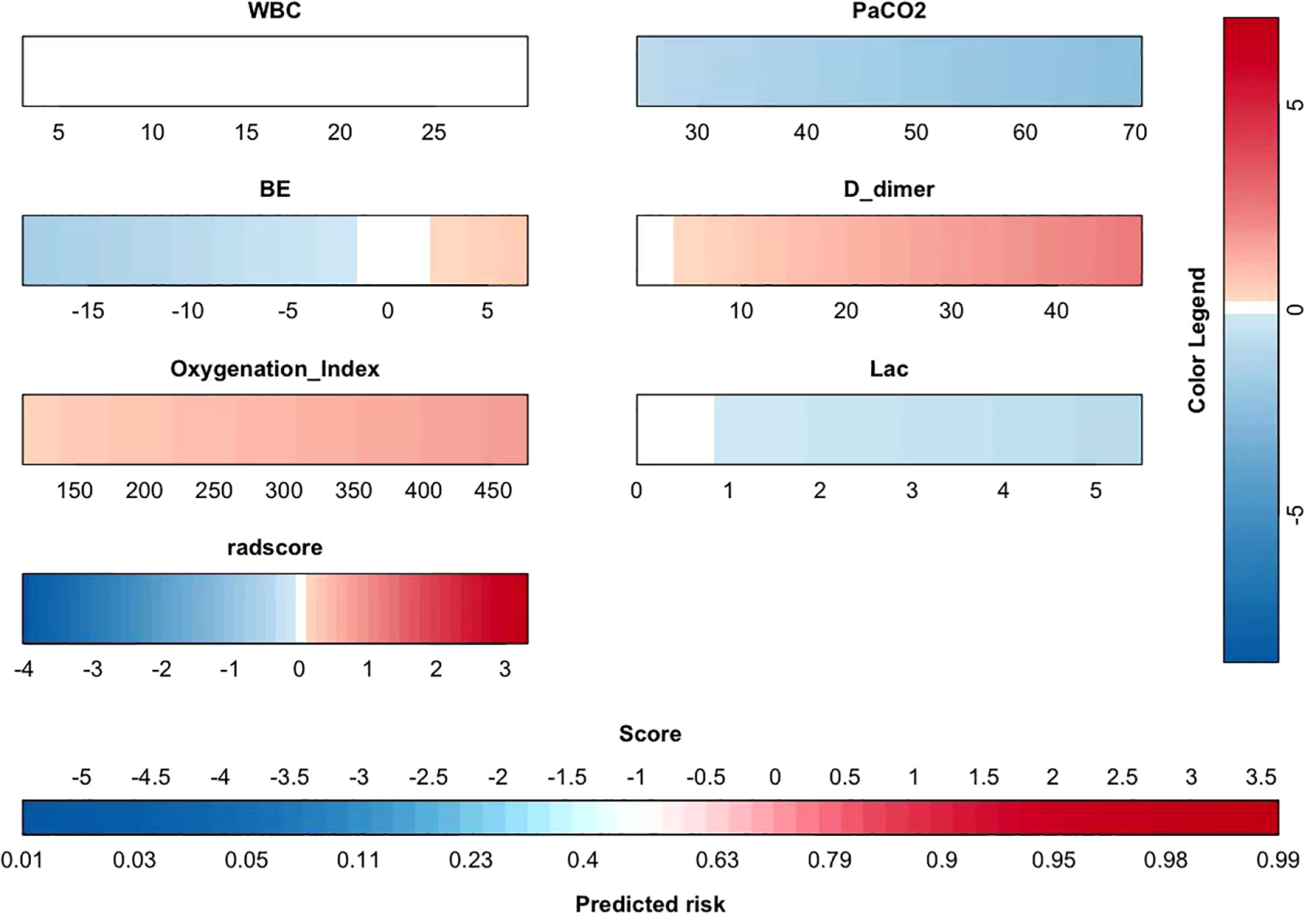
Nomogram model with radscore.

**Figure 10:**
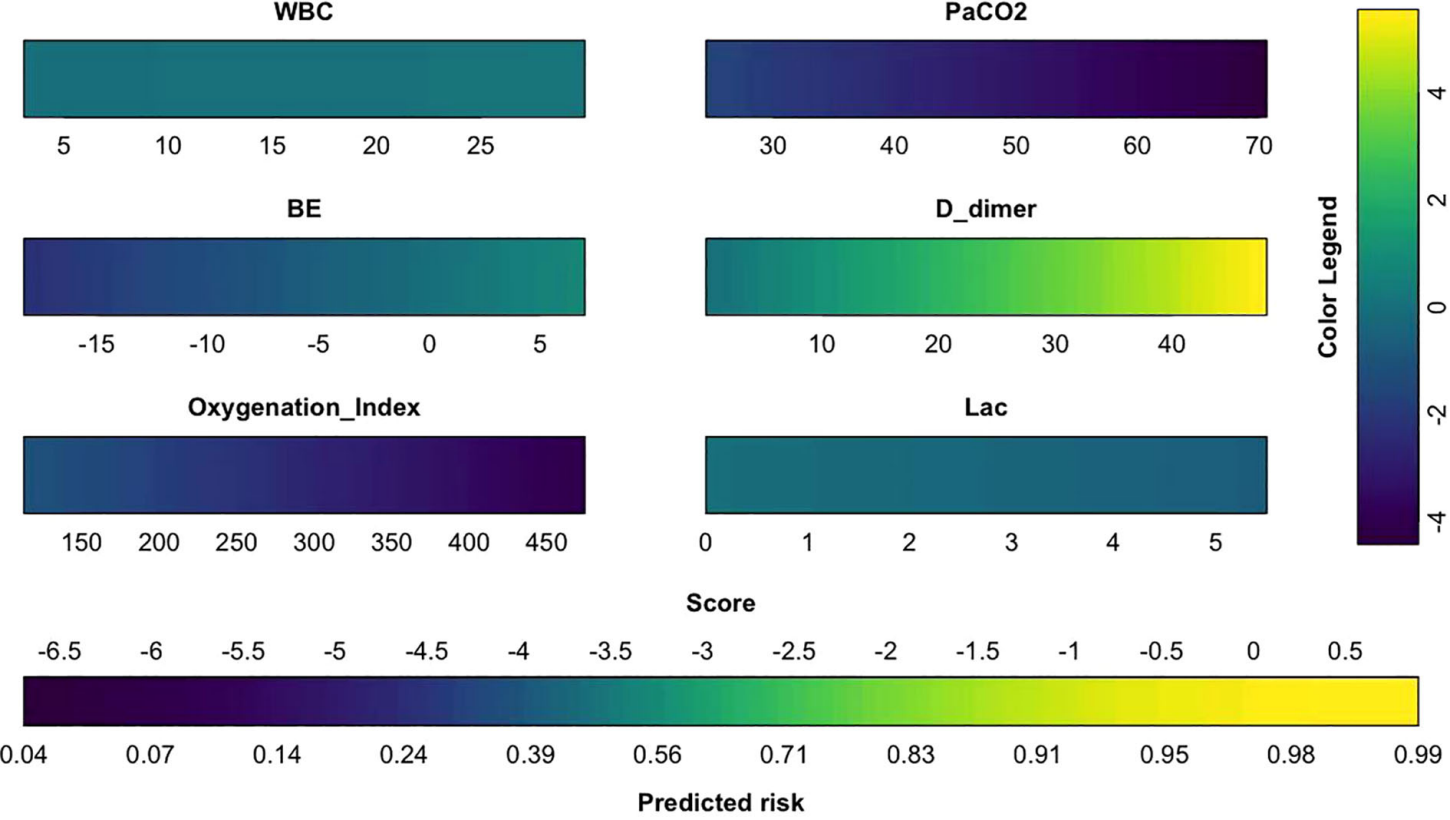
Nomogram model without radscore.

**Figure 11:**
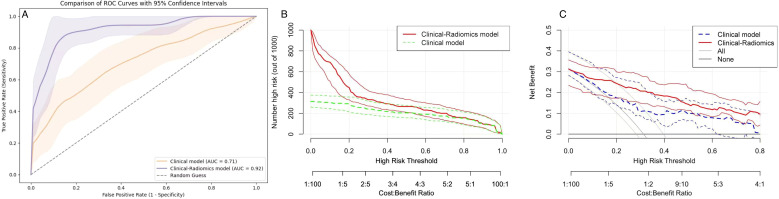
**(A)** ROC curves for two models **(B)** Decision curve analysis plot **(C)** The clinical impact curve.

The aforementioned 54 features were screened for clinical relevance using lasso regression. The top 7 statistically significant variables identified (**Figure 8**) are radscore, WBC,PaCO2,BE, D_dimer, Oxygenation Index, Lac. Then, these variables were incorporated into the construction of the nomogram model (**Figure 9**). Subsequently, a model without radscore was developed (**Figure 10**), and the accuracy and discrimination ability of the prediction models were assessed using ROC curves, with the AUC of the radscore model being 0.92 and the AUC of the model without radscore being 0.71 (**Figure 11A**). Both the DCA (Decision Curve Analysis) plot and the clinical impact curve indicate that the model with radscore outperforms the model without radscore. (**Figures 11B, C**) Furthermore, we conducted a comprehensive evaluation of the models using nine metrics: Accuracy, F1 score, AUC score, Sensitivity, Specificity, Positive Predictive Value, and Negative Predictive Value (**Table 4**).

**Table 4:** Table of performance evaluation metrics for nomogram model.

Scoring indicators	Score
Accuracy	0.925
F1score	0847
AUC score	0.916
Sensitivity	0.833
Specifity	0.955
Positive predictive value	0.862
Negative predictive value	0.945

### Machine learning algorithm comparison and verification

**Figure 12:**
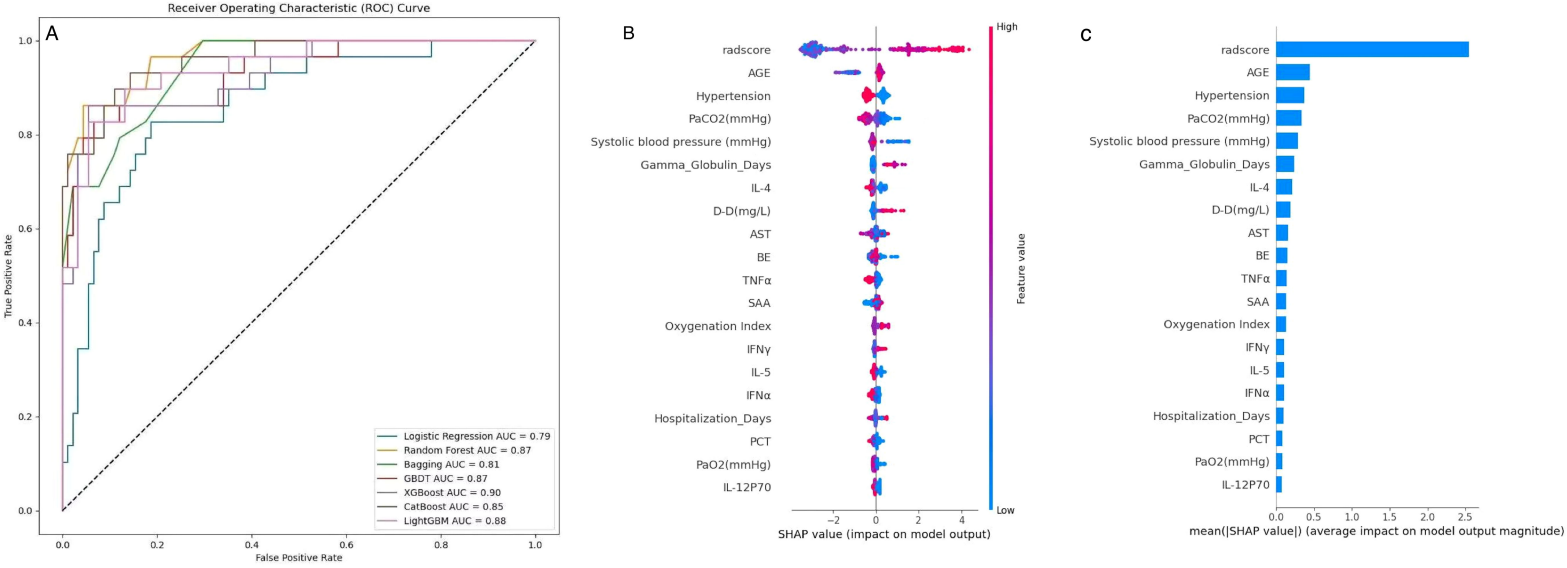
**(A)** Comparison of ROC curves in machine learning models **(B)** SHAP value analysis chart **(C)** SHAP feature importance map.

In the process of predicting 28-day mortality in SCAP patients using machine learning methods, 7 models were employed as prediction models: Logistic Regression, Random Forest, Gradient Boosting (GBDT), XGBoost (XGB), CatBoost and LightGBM. The predictive performance of various models on the test dataset was evaluated using the AUC metric (**Figure 12A**). Among them, XGB performed the best, with an AUC value of 0.90. Moreover, we also evaluated the performance of the 7 machine learning models using 7 different scoring methods, and we found that the XGB model consistently ranked among the top performers (**Table 5**).

**Table 5:** Performance evaluation metrics for machine learning model.

Model	Accuracy	F1 score	AUC score	Sensitivity	Specificity	Positive predictive value	Negative predictive value
LogisticRegression	0.825	0.667	0.791	0.724	0.857	0.618	0.907
RandomForest	0.925	0.83	0.868	0.759	0.978	0.917	0.927
Bagging	0.867	0.714	0.806	0.69	0.923	0.741	0.903
GBDT	0.908	0.807	0.869	0.793	0.945	0.821	0.935
XGBoost	0.925	0.847	0.904	0.862	0.945	0.833	0.956
CatBoost	0.9	0.786	0.852	0.759	0.945	0.815	0.925
LightGBM	0.908	0.814	0.881	0.828	0.934	0.8	0.944

### Interpretation and evaluation of the machine learning model

SHAP values are a method based on game theory, designed to explain the predictive mechanisms of machine learning models. Each SHAP value quantifies the contribution of a specific feature to a given prediction relative to the model’s average prediction. By applying the SHAP algorithm, we can rank and interpret the feature importance in the XGBoost model, as shown in (**Figures 12B, C**), the leading five features, distinguished by their SHAP values, comprise radscore,age,hypertension,PaCO2,Systolic blood pressure. Notably, the radscore ranked first, underscoring its substantial influence within the training phase of the model. This prioritization highlights the critical roles these features play in shaping the predictive accuracy and insights derived from the model.

### Traditional models in predicting mortality of SCAP patients

**Figure 13:**
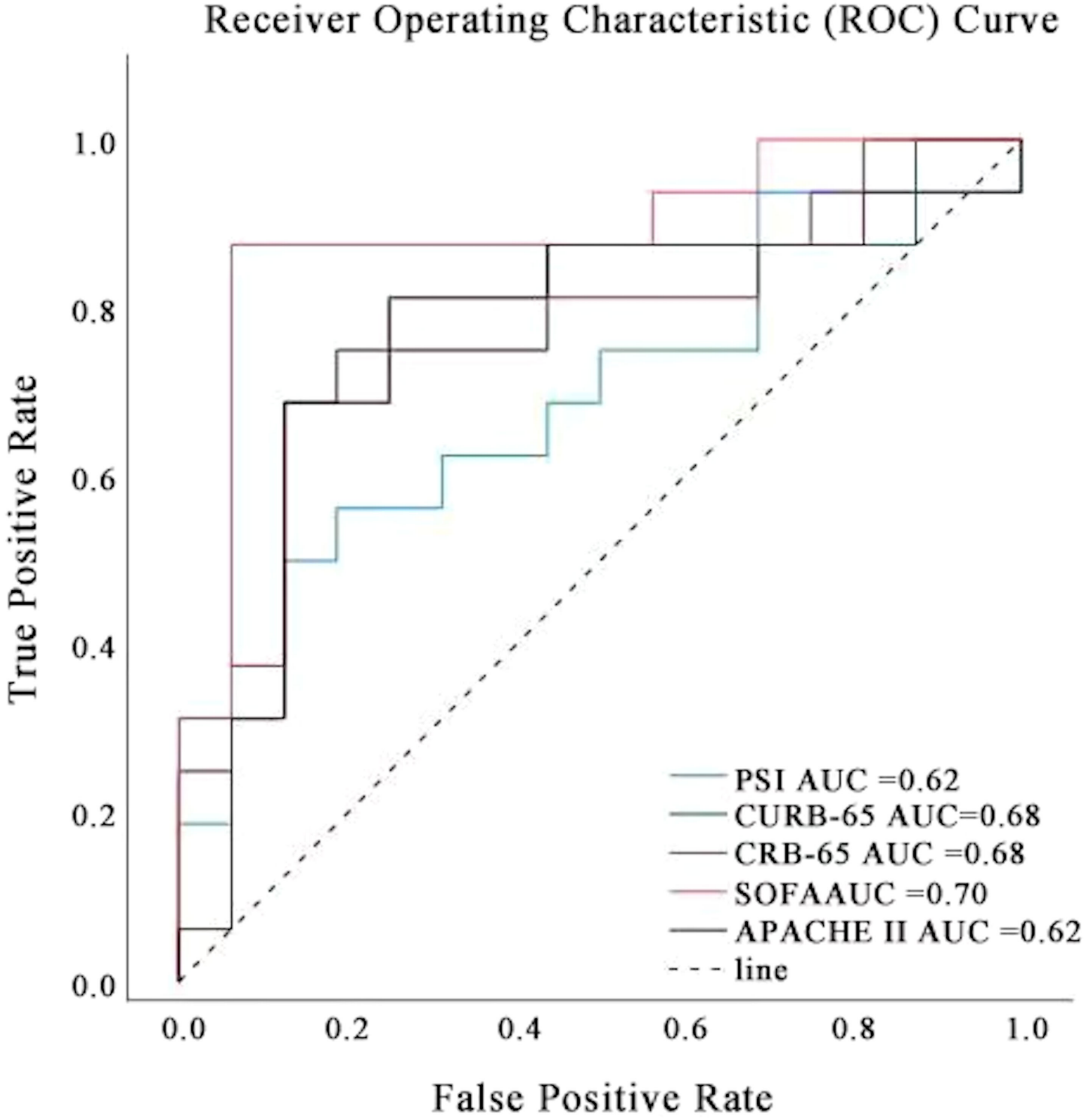
ROC curves for traditional SCAP severity scoring systems.

To further investigate the significant importance of radiomics, nomogram models, machine learning, and LLMs in assessing mortality rates among SCAP patients, we compared them with traditional pneumonia prediction models (PSI, CRB-65, CURB-65, SOFA score, APACHE II score). We plotted the ROC curves for these traditional prediction models as shown in **Figure 13**. The AUC values were 0.62 for PSI, 0.68 for both CRB-65 and CURB-65, 0.70 for the SOFA score, and 0.62 for the APACHE II score. From this, it can be seen that the predictive performance of radiomics, nomograms, machine learning, and LLMs outperforms traditional models.

### Evaluating large language models for predicting 28-day mortality in SCAP

**Figure 14:**
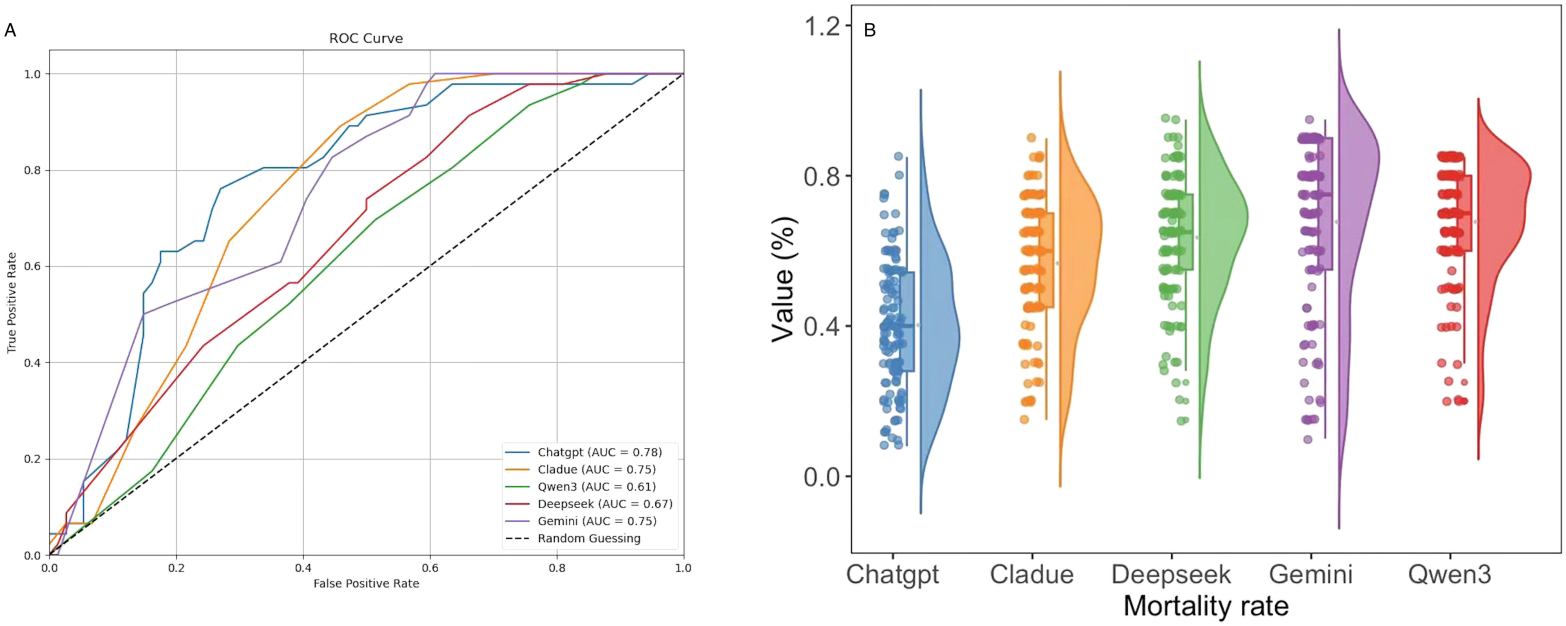
**(A)** ROC curves for five LLMs **(B)** Risk score distribution for SCAP plot.

**Figure 15:**
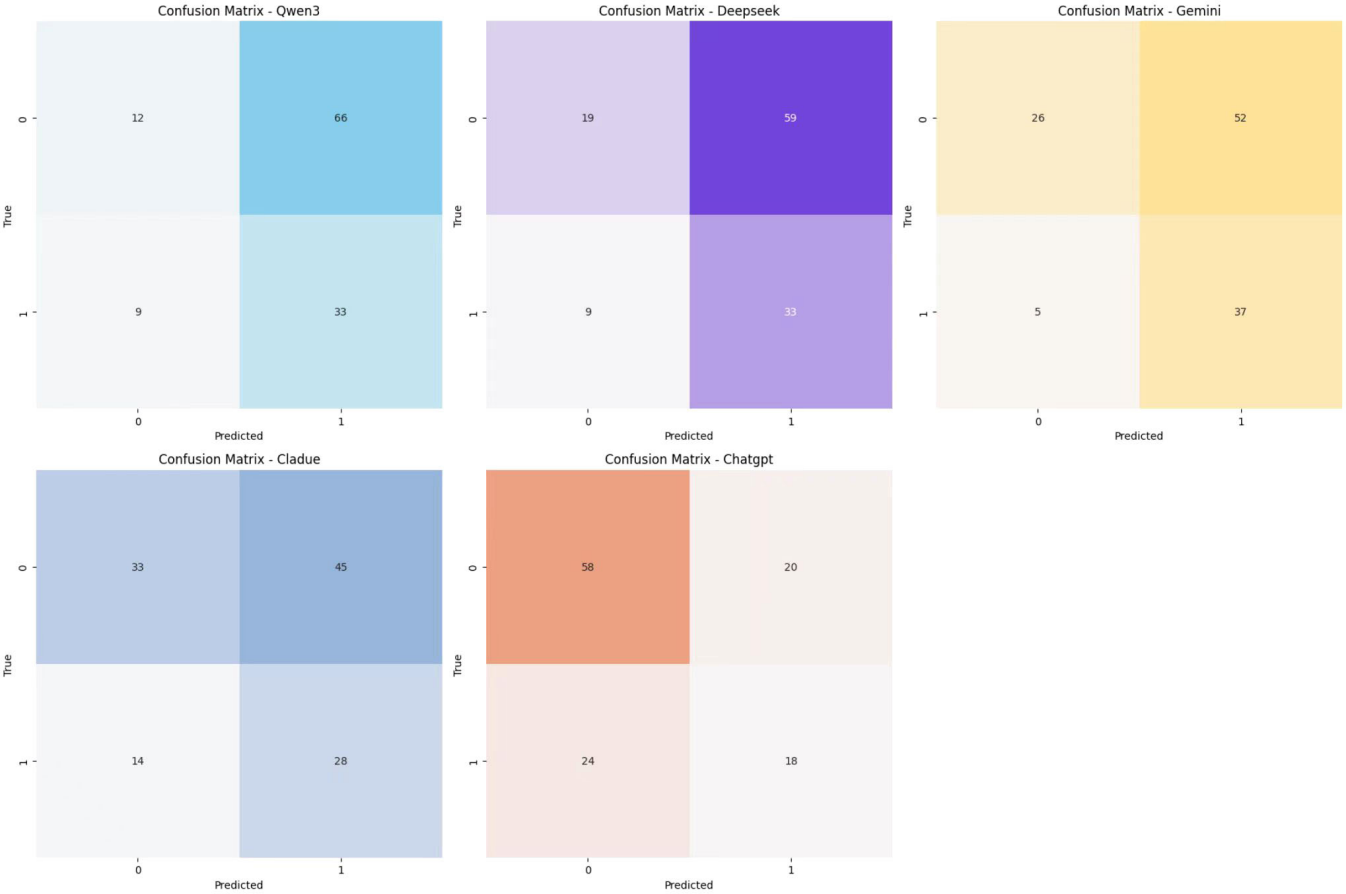
Confusion matrices comparison for the five large language models (LLMs).

This study develops a predictive system for 28-day mortality risk in patients with SCAP using five mainstream LLMs: Qwen3, DeepSeek, Gemini, Claude, and ChatGPT. The dataset of 599 patients was randomly divided into a training set and an independent test set at a 4:1 ratio. A unified medical expert role and task instruction were established to ensure consistent prediction and reasoning across all models under the same clinical context. The system integrates multidimensional patient features—including demographics, symptoms, imaging, and laboratory results—and incorporates LLM-derived prior probability predictions. By dynamically fusing data-driven modeling with expert knowledge from LLMs through a main network and a bias network, the system enhances the accuracy and reliability of mortality predictions. This figure shows the five LLMs in predicting the 28-day mortality of SCAP patients. (**Figures 14A, B**) ChatGPT has the highest AUC at 0.78, indicating strong predictive performance and an effective ability to differentiate between survivors and deaths. Gemini and Claude AUC are 0.75, close to ChatGPT, showing similarly strong predictive power. This approach demonstrates the effective application of LLMs in assisting physicians with risk prediction for patients with SCAP, providing valuable support and insights for clinical decision-making. **Figure 15** presents the confusion matrices of five models on a binary classification task, used to evaluate their classification performance. Here, label “0” represents death, and label “1” represents survival. In each matrix, the first column indicates samples with the actual label 0, where the first row shows the number correctly predicted (i.e., true negatives), and the second row shows the number incorrectly predicted as 1 (i.e., false positives). The second column represents samples with the actual label 1, where the first row indicates the number incorrectly predicted as 0 (i.e., false negatives), and the second row shows the number correctly predicted (i.e., true positives). The specific data are as follows: for the Qwen3 model, the true negatives, false positives, false negatives, and true positives are 12, 66, 9, and 33, respectively; for the Deepseek model, they are 19, 59, 9, and 33; for the Gemini model, they are 26, 52, 5, and 37; for the Cladue model, they are 33, 45, 14, and 28; and for the Chatgpt model, they are 58, 20, 24, and 18.Chatgpt showed the highest number of correct predictions.

## Discussion

This study integrates radiomics, ML, and LLMs to predict 28-day mortality in SCAP patients, offering a novel approach to clinical risk stratification. Our findings highlight the potential of these advanced computational techniques in improving early diagnosis, prognosis prediction, and personalized treatment in SCAP management.

A key contribution of this study is the identification of clinically significant predictors of SCAP mortality, including radscore, inflammatory markers (CRP, PCT, IL-6, IL-8, TNF-α), and oxygenation index (PaO2/FiO2). These findings align with prior research demonstrating that systemic inflammation and hypoxia play central roles in SCAP progression and patient outcomes. Our results show that radiomics-based radscore was the most influential predictor of SCAP mortality in ML models, reinforcing the role of quantitative imaging in pneumonia assessment. Unlike traditional imaging that relies on subjective interpretation, radiomics enables early detection of lung abnormalities associated with disease severity, improving ICU triage decisions and guiding aggressive interventions for high-risk patients. The inclusion of these markers in predictive models supports real-time risk assessment, allowing clinicians to implement targeted anti-inflammatory therapies in critically ill SCAP patients. Similarly, a low PaO2/FiO2 ratio was confirmed as a strong predictor of mortality, indicative of respiratory failure and severe hypoxemia, emphasizing the necessity for early oxygen therapy, non-invasive ventilation, or mechanical ventilation strategies to enhance survival outcomes in high-risk SCAP patients. Traditional SCAP severity scoring systems, such as PSI (Pneumonia Severity Index) and CURB-65, provide a baseline risk assessment but may oversimplify complex disease dynamics. Our XGB machine learning model (AUC: 0.90) and Clinical-Radiomics model (AUC: 0.92) exhibited superior predictive performance compared to conventional scoring methods, suggesting that integrating multi-source clinical and imaging data enhances risk stratification. To rigorously dissect the sources of this performance improvement, we compared the models across three levels of complexity. First, traditional scoring systems (PSI, CURB-65) achieved baseline AUCs ranging from 0.62 to 0.68. Second, our machine learning model trained solely on clinical variables (Clinical Model) reached an AUC of 0.71, indicating that advanced algorithms can extract superior predictive value from clinical data compared to static rule-based scores. Crucially, however, the most significant performance leap was observed only after integrating radiomics features (Clinical-Radiomics Model, AUC 0.92). This comparison suggests that while the ML algorithm contributes a moderate performance gain via non-linear modeling, the primary driver of the superior accuracy in our proposed framework is the inclusion of high-dimensional radiomic features, which capture sub-visual lung pathology often missed by conventional clinical assessments.

Radiomics, by extracting high-dimensional imaging features from CT scans, provides a new perspective on the severity and prognosis of SCAP, significantly enhancing the performance of mortality prediction models compared to traditional clinical data. These features not only reflect morphological changes in lung lesions but also combine multidimensional information to help accurately identify high-risk patients, demonstrating the potential of radiomics in quantifying disease severity and assisting decision-making. The Nomogram is an intuitive predictive tool that, when combined with radiomics and machine learning models, can enhance prediction accuracy. Although it has good practical utility in clinical settings, it also has some limitations. First, it is susceptible to overfitting and requires a large amount of high-quality, representative data. Second, the Nomogram typically assumes linear relationships between variables, overlooking the possibility of non-linear and interaction effects. Additionally, the lack of dynamic updates and the ability to adapt to complex clinical environments may cause it to become ineffective over the long term. Finally, the difficulty of external validation and the need for clinical doctors to understand the model also limit its widespread application (44). Machine learning algorithms, including random forests, support vector machines, and deep neural networks, provide effective tools for precise mortality prediction by combining multiple features. However, machine learning faces multiple challenges in clinical applications, including mismatches between laboratory conditions and real-world environments during development and validation, lack of external validation, data gaps and distribution issues, high costs of data integration, privacy and security risks, limited model transparency and interpretability, and distrust of “black-box” models by healthcare professionals. Regarding this interpretability challenge, while SHAP analysis provides intuitive insights into feature importance, we acknowledge its theoretical limitations in high-dimensional contexts. Specifically, SHAP values may not guarantee unique explanations when features exhibit high multicollinearity, as credit can be arbitrarily split among redundant variables. In this study, we proactively addressed this issue by employing Lasso regression as a feature selection filter prior to modeling. By selecting representative features from correlated groups, Lasso effectively reduced multicollinearity, thereby enhancing the stability and credibility of the subsequent SHAP explanations. Nevertheless, the resulting feature importance should be interpreted within the context of this optimized subset. Additionally, incomplete regulatory frameworks, insufficient compatibility with existing healthcare systems, difficulties in integrating complex technologies into busy workflows, and unclear clinical problem definitions further limit its application. To realize its potential, collaborative efforts are needed to address these challenges and ensure effectiveness, safety, and clinical value (45). Lastly, the introduction of LLMs offers a novel approach by integrating unstructured textual data from electronic health records, imaging reports, and lab results, enabling the discovery of semantic associations that enhance understanding of disease progression. While LLMs effectively assist in predicting SCAP mortality, challenges such as data privacy, model generalization, and potential biases need further exploration to validate their applicability across different datasets and populations (39, 46–48). Specifically, regarding these biases, it is worth noting that while the SMOTE technique was employed during training to mitigate class imbalance, the confusion matrices on the independent test set (**Figure 15**) still reveal varying degrees of class bias among different LLMs. Some models exhibited a tendency to favor the majority class (survival). This observation underscores the limitation of relying solely on standalone LLMs for prognosis and highlights the necessity of our fusion strategy, where SMOTE-trained machine learning features effectively compensate for these biases, ensuring robust performance across both classes.

Ethically, the integration of LLMs into clinical workflows necessitates a rigorous framework to ensure safety and trust. First, regarding data privacy, strict de-identification protocols must be enforced, and the deployment of local, on-premise LLMs (e.g., localized versions of Qwen or LLaMA) is recommended to prevent sensitive patient data from traversing external servers. Second, regarding transparency, our dual-driven approach mitigates the ‘black-box’ risk by offering interpretability through two channels: SHAP values for quantitative feature contribution and LLM-generated textual reasoning for qualitative logic. Finally, concerning accountability, we emphasize that such predictive systems are designed as decision support tools within a ‘Human-in-the-Loop’ paradigm. The ultimate clinical decision and responsibility reside with the physician, who must validate the AI’s recommendations against their professional judgment and the patient’s specific context.

The integration of ML and LLMs in SCAP management could enable personalized treatment pathways, with applications in early risk stratification, therapy optimization, and real-time clinical decision support. By combining radiomics, laboratory biomarkers, and clinical scores, an automated early warning system could be developed to identify high-risk patients upon hospital admission. Personalized risk profiles could further assist in optimizing antibiotic regimens, anti-inflammatory therapies, and respiratory support strategies, ensuring tailored interventions. Additionally, LLMs could enhance clinical decision support systems (CDSS) by enabling automated interpretation of electronic medical records (EMRs), assisting physicians in real-time diagnosis and treatment decisions. Despite these promising applications, certain limitations must be acknowledged. First, although this study benefited from a dual-center design which offers better generalizability than single-center cohorts, potential geographic biases and overfitting risks cannot be entirely ruled out. While we employed Lasso regression to reduce feature redundancy and strictly evaluated performance on an independent test set, the model’s robustness across broader populations remains to be verified. Therefore, future work must prioritize large-scale, multi-center external validation, particularly prospective studies across diverse healthcare settings, to fully confirm the model’s clinical utility and generalizability. Furthermore, while our conservative feature selection approach improved model interpretability, future research should investigate deep learning models capable of handling larger feature sets.

## Conclusion

This study demonstrates the potential of radiomics, Nomogram, machine learning, and large language models in predicting mortality in severe community-acquired pneumonia and highlights the importance of integrating multi-source data. In the future, with the increase in data volume and advancements in algorithms, these technologies are expected to provide more precise and interpretable decision support for the personalized management of patients with SCAP.

## Data Availability

The datasets presented in this study can be found in online repositories. The names of the repository/repositories and accession number(s) can be found in the article/supplementary material.

## References

[1] Anderson R Feldman C . The global burden of community-acquired pneumonia in adults, encompassing invasive pneumococcal disease and the prevalence of its associated cardiovascular events, with a focus on pneumolysin and macrolide antibiotics in pathogenesis and therapy. Int J Mol Sci. (2023) 24:11038. doi: 10.3390/ijms241311038, PMID: 37446214 PMC10341596

[2] Jiang N Long QY Zheng YL Gao ZC . Advances in epidemiology, etiology, and treatment of community-acquired pneumonia. Zhonghua Yu Fang Yi Xue Za Zhi. (2023) 57:91–9. doi: 10.3760/cma.j.cn112150-20220308-00214, PMID: 36655264

[3] Oliveira ESPG Cerqueira Batista Filho LA Ismael PF Victoria VES Alexandre TM Larissa SM . Community-acquired pneumonia: Epidemiology, diagnosis, prognostic severity scales, and new therapeutic options. Medwave. (2023) 23:e2719. doi: 10.5867/medwave.2023.11.2719, PMID: 38091488

[4] Marti C Garin N Grosgurin O Poncet A Combescure C Carballo S . Prediction of severe community-acquired pneumonia: a systematic review and meta-analysis. Crit Care. (2012) 16:R141. doi: 10.1186/cc11447, PMID: 22839689 PMC3580727

[5] Charles PG Wolfe R Whitby M Fine MJ Fuller AJ Stirling R . SMART-COP: a tool for predicting the need for intensive respiratory or vasopressor support in community-acquired pneumonia. Clin Infect Dis. (2008) 47:375–84. doi: 10.1086/589754, PMID: 18558884

[6] Mandell LA Wunderink RG Anzueto A Bartlett JG Campbell GD Dean NC . Infectious Diseases Society of America/American Thoracic Society consensus guidelines on the management of community-acquired pneumonia in adults. Clin Infect Dis. (2007) 44 Suppl 2:S27–72. doi: 10.1086/511159, PMID: 17278083 PMC7107997

[7] Capelastegui A España PP Quintana JM Areitio I Gorordo I Egurrola M . Validation of a predictive rule for the management of community-acquired pneumonia. Eur Respir J. (2006) 27:151–7. doi: 10.1183/09031936.06.00062505, PMID: 16387948

[8] Niederman MS Mandell LA Anzueto A Bass JB Broughton WA Campbell GD . Guidelines for the management of adults with community-acquired pneumonia. Diagnosis, assessment of severity, antimicrobial therapy, and prevention. Am J Respir Crit Care Med. (2001) 163:1730–54. doi: 10.1164/ajrccm.163.7.at1010, PMID: 11401897

[9] Fine MJ Auble TE Yealy DM Hanusa BH Weissfeld LA Singer DE . A prediction rule to identify low-risk patients with community-acquired pneumonia. N Engl J Med. (1997) 336:243–50. doi: 10.1056/NEJM199701233360402, PMID: 8995086

[10] Lambin P Leijenaar RTH Deist TM Peerlings J de Jong EEC van Timmeren J . Radiomics: the bridge between medical imaging and personalized medicine. Nat Rev Clin Oncol. (2017) 14:749–62. doi: 10.1038/nrclinonc.2017.141, PMID: 28975929

[11] Gillies RJ Kinahan PE Hricak H . Radiomics: images are more than pictures, they are data. Radiology. (2016) 278:563–77. doi: 10.1148/radiol.2015151169, PMID: 26579733 PMC4734157

[12] Aerts HJ Velazquez ER Leijenaar RT Parmar C Grossmann P Carvalho S . Decoding tumor phenotype by noninvasive imaging using a quantitative radiomics approach. Nat Commun. (2014) 5:4006. doi: 10.1038/ncomms5006, PMID: 24892406 PMC4059926

[13] Lambin P van Stiphout RG Starmans MH Rios-Velazquez E Nalbantov G Aerts HJ . Predicting outcomes in radiation oncology–multifactorial decision support systems. Nat Rev Clin Oncol. (2013) 10:27–40. doi: 10.1038/nrclinonc.2012.196, PMID: 23165123 PMC4555846

[14] Lambin P Rios-Velazquez E Leijenaar R Carvalho S van Stiphout RG Granton P . Radiomics: extracting more information from medical images using advanced feature analysis. Eur J Cancer. (2012) 48:441–6. doi: 10.1016/j.ejca.2011.11.036, PMID: 22257792 PMC4533986

[15] Schoolman HM Bernstein LM . Computer use in diagnosis, prognosis, and therapy. Science. (1978) 200:926–31. doi: 10.1126/science.347580, PMID: 347580

[16] Zhao C Xu Y He Z Tang JS Zhang YJ Han JG . Lung segmentation and automatic detection of COVID-19 using radiomic features from chest CT images. Pattern Recognit. (2021) 119:108071. doi: 10.1016/j.patcog.2021.108071, PMID: 34092815 PMC8169223

[17] Zhang XG Wang DW Shao J Tian S Tan WX Ma Y . A deep learning integrated radiomics model for identification of coronavirus disease 2019 using computed tomography. Sci Rep. (2021) 11:3938. doi: 10.1038/s41598-021-83237-6, PMID: 33594159 PMC7886892

[18] Wang B Li M Ma H Han FF Wang Y Zhao SY . Computed tomography-based predictive nomogram for differentiating primary progressive pulmonary tuberculosis from community-acquired pneumonia in children. BMC Med Imaging. (2019) 19:63. doi: 10.1186/s12880-019-0355-z, PMID: 31395012 PMC6688341

[19] Balbinot F da Costa Batista Guedes Á Nascimento DZ Zampieri JF Alves GR Marchiori E . Advances in imaging and automated quantification of pulmonary diseases in non-neoplastic diseases. Lung. (2016) 194:871–9. doi: 10.1007/s00408-016-9940-x, PMID: 27663257

[20] Iasonos A Schrag D Raj GV Panageas KS . How to build and interpret a nomogram for cancer prognosis. J Clin Oncol. (2008) 26:1364–70. doi: 10.1200/JCO.2007.12.9791, PMID: 18323559

[21] Li Z Kang S Kang H . Development and validation of nomograms for predicting cardiovascular disease risk in patients with prediabetes and diabetes. Sci Rep. (2024) 14:20909. doi: 10.1038/s41598-024-71904-3, PMID: 39245747 PMC11381537

[22] Bonkhoff AK Grefkes C . Precision medicine in stroke: towards personalized outcome predictions using artificial intelligence. Brain. (2022) 145:457–75. doi: 10.1093/brain/awab439, PMID: 34918041 PMC9014757

[23] Chen XX Wang XM Zhang K Fung KM Thai TC Moore K . Recent advances and clinical applications of deep learning in medical image analysis. Med Image Anal. (2022) 79:102444. doi: 10.1016/j.media.2022.102444, PMID: 35472844 PMC9156578

[24] Ching T Himmelstein DS Beaulieu-Jones BK Kalinin AA Do BT Way GP . Opportunities and obstacles for deep learning in biology and medicine. J R Soc Interface. (2018) 15:20170387. doi: 10.1098/rsif.2017.0387, PMID: 29618526 PMC5938574

[25] Eckhardt CM Madjarova SJ Williams RJ Ollivier M Karlsson J Pareek A . Unsupervised machine learning methods and emerging applications in healthcare. Knee Surg Sports Traumatol Arthrosc. (2023) 31:376–81. doi: 10.1007/s00167-022-07233-7, PMID: 36378293

[26] Gonem S Janssens W Das N Topalovic M . Applications of artificial intelligence and machine learning in respiratory medicine. Thorax. (2020) 75:695–701. doi: 10.1136/thoraxjnl-2020-214556, PMID: 32409611

[27] Krishnan R Rajpurkar P Topol EJ . Self-supervised learning in medicine and healthcare. Nat BioMed Eng. (2022) 6:1346–52. doi: 10.1038/s41551-022-00914-1, PMID: 35953649

[28] Mayampurath A Ajith A Anderson-Smits C Chang SC Brouwer E Johnson J . Early diagnosis of primary immunodeficiency disease using clinical data and machine learning. J Allergy Clin Immunol Pract. (2022) 10:3002–7.e5. doi: 10.1016/j.jaip.2022.08.041, PMID: 36108921

[29] Myszczynska MA Ojamies PN Lacoste AMB Neil D Saffari A Mead R . Applications of machine learning to diagnosis and treatment of neurodegenerative diseases. Nat Rev Neurol. (2020) 16:440–56. doi: 10.1038/s41582-020-0377-8, PMID: 32669685

[30] Peiffer-Smadja N Rawson TM Ahmad R Buchard A Georgiou P Lescure FX . Machine learning for clinical decision support in infectious diseases: a narrative review of current applications. Clin Microbiol Infect. (2020) 26:584–95. doi: 10.1016/j.cmi.2019.09.009, PMID: 31539636

[31] Segar MW Pandey A . Omics, machine learning, and personalized medicine in heart failure with preserved ejection fraction: promising future or false hope? Eur J Heart Fail. (2021) 23:992–4. doi: 10.1002/ejhf.2246, PMID: 34021682

[32] Subrahmanya SVG Shetty DK Patil V Hameed BMZ Paul R Smriti K . The role of data science in healthcare advancements: applications, benefits, and future prospects. Ir J Med Sci. (2022) 191:1473–83. doi: 10.1007/s11845-021-02730-z, PMID: 34398394 PMC9308575

[33] Gong EJ Bang CS Lee JJ Baik GH Lim H Jeong JH . Deep learning-based clinical decision support system for gastric neoplasms in real-time endoscopy: development and validation study. Endoscopy. (2023) 55:701–8. doi: 10.1055/a-2031-0691, PMID: 36754065

[34] Issa NT Stathias V Schürer S Dakshanamurthy S . Machine and deep learning approaches for cancer drug repurposing. Semin Cancer Biol. (2021) 68:132–42. doi: 10.1016/j.semcancer.2019.12.011, PMID: 31904426 PMC7723306

[35] van Veen D van Uden C Blankemeier L Delbrouck JB Aali A Bluethgen C . Adapted large language models can outperform medical experts in clinical text summarization. Nat Med. (2024) 30:1134–42. doi: 10.1038/s41591-024-02855-5, PMID: 38413730 PMC11479659

[36] Wei JH Zhuo LL Fu XZ Zeng XX Wang L Zou Q . DrugReAlign: a multisource prompt framework for drug repurposing based on large language models. BMC Biol. (2024) 22:226. doi: 10.1186/s12915-024-02028-3, PMID: 39379930 PMC11463036

[37] Yuan QM Tian C Song YD Ou PH Zhu MM Zhao HY . GPSFun: geometry-aware protein sequence function predictions with language models. Nucleic Acids Res. (2024) 52:W248–w55. doi: 10.1093/nar/gkae381, PMID: 38738636 PMC11223820

[38] Yang X Chen AK PourNejatian N Shin HC Smith KE Parisien C . A large language model for electronic health records. NPJ Digit Med. (2022) 5:194. doi: 10.1038/s41746-022-00742-2, PMID: 36572766 PMC9792464

[39] Zhang HY Zhou Y Zhang ZC Sun HC Pan ZQ Mou MJ . Large language model-based natural language encoding could be all you need for drug biomedical association prediction. Anal Chem. (2024) 96:12395–403. doi: 10.1021/acs.analchem.4c01793, PMID: 39011990

[40] Benary M Wang XD Schmidt M Soll D Hilfenhaus G Nassir M . Leveraging large language models for decision support in personalized oncology. JAMA Netw Open. (2023) 6:e2343689. doi: 10.1001/jamanetworkopen.2023.43689, PMID: 37976064 PMC10656647

[41] Chawla KWB NV Hall LO Kegelmeyer WP . SMOTE: synthetic minority over-sampling technique. J Artif Intell Res. (2002) 16:321–57. doi: 10.1613/jair.953

[42] Merrick L Taly A . The explanation game: explaining machine learning models using shapley values. Mach Learn Knowledge Extraction. (2020), 17–38. doi: 10.1007/978-3-030-57321-8_2

[43] Lundberg SM Lee S-I . A unified approach to interpreting model predictions. Adv Neural Inf Process Syst. (2017) 30:1–10.

[44] Lee W Lam S-K Zhang Y Yang R Cai J . Review of methodological workflow, interpretation and limitations of nomogram application in cancer study. Radiat Med Prot. (2022) 3:200–7. doi: 10.1016/j.radmp.2022.08.004

[45] Nilius H Tsouka S Nagler M Masoodi M . Machine learning applications in precision medicine: Overcoming challenges and unlocking potential. TrAC Trends Analytical Chem. (2024) 179:117872. doi: 10.1016/j.trac.2024.117872

[46] Ong JCL Chang SY William W Butte AJ Shah NH Chew LST . Ethical and regulatory challenges of large language models in medicine. Lancet Digit Health. (2024) 6:e428–e32. doi: 10.1016/S2589-7500(24)00061-X, PMID: 38658283

[47] Yang R Tan TF Lu W Thirunavukarasu AJ Ting DSW Liu N . Large language models in health care: Development, applications, and challenges. Health Care Sci. (2023) 2:255–63. doi: 10.1002/hcs2.61, PMID: 38939520 PMC11080827

[48] AlSaad R Abd-Alrazaq A Boughorbel S Ahmed A Renault MA Damseh R . Multimodal large language models in health care: applications, challenges, and future outlook. J Med Internet Res. (2024) 26:e59505. doi: 10.2196/59505, PMID: 39321458 PMC11464944

