# Correction: Comparing the performance of radiomics, nomograms, machine learning, and large language models in predicting 28-day mortality in severe community-acquired pneumonia patients

**DOI:** 10.3389/fimmu.2026.1802895

**Published:** 2026-03-19

**Authors:** Tingting Lin, Huimin Wan, Yifei Liang, Jie Ming, Jingjing Lu, Zhongliang Guo

**Affiliations:** 1 Fujian Medical University Xiamen Hong’ai Hospital, Xiamen, China; 2 Shanghai East Hospital, School of Medicine, Tongji University, Shanghai, China

**Keywords:** LLMS, machine learning, nomogram, radiomics, SCAP

Affiliation “Fujian Medical University Xiamen Hong’ai Hospital, Xiamen, China” was erroneously given as “Fujian Medical University, Xiamen Humanity Hospital, Xiamen, China”.

The original version of this article has been updated.

